# DNA mismatch repair protects the genome from oxygen-induced replicative mutagenesis

**DOI:** 10.1093/nar/gkad775

**Published:** 2023-10-04

**Authors:** Rita Lózsa, Eszter Németh, Judit Z Gervai, Bence G Márkus, Sándor Kollarics, Zsolt Gyüre, Judit Tóth, Ferenc Simon, Dávid Szüts

**Affiliations:** Institute of Enzymology, Research Centre for Natural Sciences, H-1117 Budapest, Hungary; Institute of Enzymology, Research Centre for Natural Sciences, H-1117 Budapest, Hungary; Institute of Enzymology, Research Centre for Natural Sciences, H-1117 Budapest, Hungary; Stavropoulos Center for Complex Quantum Matter, Department of Physics and Astronomy, University of Notre Dame, Notre Dame, IN 46556, USA; Institute for Solid State Physics and Optics, Wigner Research Centre for Physics, H-1525 Budapest, Hungary; Department of Physics, Institute of Physics, Budapest University of Technology and Economics, H-1111 Budapest, Hungary; Institute for Solid State Physics and Optics, Wigner Research Centre for Physics, H-1525 Budapest, Hungary; Department of Physics, Institute of Physics, Budapest University of Technology and Economics, H-1111 Budapest, Hungary; Institute of Enzymology, Research Centre for Natural Sciences, H-1117 Budapest, Hungary; Doctoral School of Molecular Medicine, Semmelweis University, H-1085 Budapest, Hungary; Turbine Simulated Cell Technologies, H-1027 Budapest, Hungary; Institute of Enzymology, Research Centre for Natural Sciences, H-1117 Budapest, Hungary; Department of Applied Biotechnology and Food Science, Budapest University of Technology and Economics, H-1111 Budapest, Hungary; Institute for Solid State Physics and Optics, Wigner Research Centre for Physics, H-1525 Budapest, Hungary; Department of Physics, Institute of Physics, Budapest University of Technology and Economics, H-1111 Budapest, Hungary; Institute of Enzymology, Research Centre for Natural Sciences, H-1117 Budapest, Hungary; National Laboratory for Drug Research and Development, H-1117 Budapest, Hungary

## Abstract

DNA mismatch repair (MMR) corrects mismatched DNA bases arising from multiple sources including polymerase errors and base damage. By detecting spontaneous mutagenesis using whole genome sequencing of cultured MMR deficient human cell lines, we show that a primary role of MMR is the repair of oxygen-induced mismatches. We found an approximately twofold higher mutation rate in MSH6 deficient DLD-1 cells or MHL1 deficient HCT116 cells exposed to atmospheric conditions as opposed to mild hypoxia, which correlated with oxidant levels measured using electron paramagnetic resonance spectroscopy. The oxygen-induced mutations were dominated by T to C base substitutions and single T deletions found primarily on the lagging strand. A broad sequence context preference, dependence on replication timing and a lack of transcriptional strand bias further suggested that oxygen-induced mutations arise from polymerase errors rather than oxidative base damage. We defined separate low and high oxygen–specific MMR deficiency mutation signatures common to the two cell lines and showed that the effect of oxygen is observable in MMR deficient cancer genomes, where it best correlates with the contribution of mutation signature SBS21. Our results imply that MMR corrects oxygen-induced genomic mismatches introduced by a replicative process in proliferating cells.

## Graphical Abstract

**Graphical Abstract:**
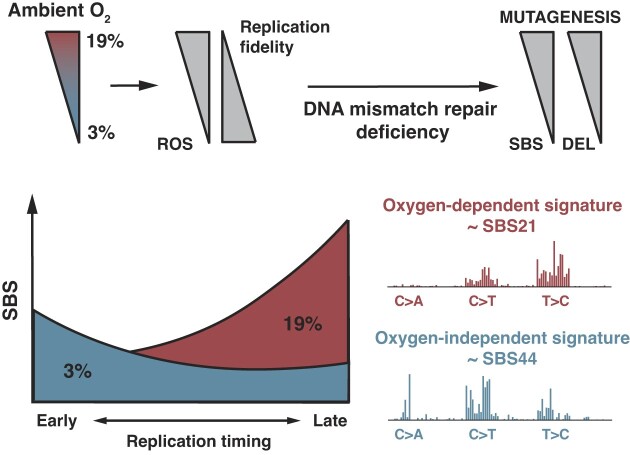

## Introduction

DNA mismatch repair (MMR) is a conserved DNA repair process that detects and corrects base-base mismatches and short bulged loops in the double stranded helix of genomic DNA. The primary role of MMR is the correction of mistakes produced by replicative DNA polymerases as they copy the template DNA strand (1,2). Cancers with inactivating defects in MMR genes show a very high burden of somatic base substitution mutations, accompanied by insertions/deletions (indels) at homopolymeric stretches or other short repeat sequences, an indel phenomenon termed microsatellite instability (3,4). Genomic evidence of MMR deficiency is used for treatment selection (5,6), and an improved understanding of the mutagenic mechanisms behind the predictive biomarkers may help patient stratification.

DNA strand mismatches are recognised by the conserved MutSα and MutSβ complexes in human cells, with overlapping specificities. MutSα, a heterodimer of MSH2 and MSH6, binds both base mismatches and 1-base insertion-deletion loops; whereas the MSH2-MSH3 heterodimer of MutSβ has low affinity for mispaired bases and shows preference for insertion-deletion loops that are several bases in length (7,8). The MutLα dimer of MLH1 and PMS2 joins either MutS complex on DNA to perform a strand-specific incision in the vicinity of the mismatch. Exonuclease 1 (EXO1) is recruited to excise the newly synthesised DNA strand, and the resulting gap is filled by a replicative DNA polymerase.

Mutagenesis in MMR deficient (MMRd) cells gives a direct view of primary mutagenic processes that provide substrates for MMR, and hints at different functions of MMR. The largest datasets are derived from cancer genomics studies, and non-supervised extraction of mutational signatures can be used to interpret the mutational spectra and subdivide the mutational sets by underlying biological causes (9,10). Up to seven different single base substitution (SBS) signatures have been attributed to MMR deficiency in cancer cells (11), suggesting a great complexity of mutagenic processes. We previously presented a targeted analysis of MMRd mutations that produced only two mutational signatures which adequately reconstitute SBS mutagenesis in MMRd tumour samples (12) and likely represent independent biological processes. One of the extracted signatures (MMRd-A) is dominated by C > T mutations at CpG sites, which have been attributed to two distinct mechanisms both related to CpG methylation: the higher error rate of the replicative polymerase at 5-methylcytosine (5mC) versus cytosine bases (13), and the replication-independent repair of G:T mismatches resulting from the spontaneous deamination of 5mC, which involves MutSα but not MutLα (14). The spectrum of the second signature (MMRd-B) is dominated by T > C, C > T transition and C > A transversion mutations, where each category includes mutations on the complementary strand (12,14). MMRd-B shows greatest similarity to MMRd-related COSMIC signatures SBS26 and SBS44 (11), which were also confirmed in MMRd tumour genomes by a large recent study (15).

Transition mutagenesis in MMRd cells may largely result from replication errors, as T:G mismatches are the most thermodynamically stable (16), and misincorporation of T opposite G or G opposite T would cause C > T and T > C mutations, respectively. Indeed, the same mutation classes further increase with the inactivation of the proofreading function of replicative polymerases in MMRd yeast cells (17). However, the *in vitro* error spectra of exonuclease deficient human Pol ϵ and Pol δ show comparable rates of various transition and transversion mutation classes with marked differences between the two enzymes (18,19), suggesting that the replication-dependent SBS spectrum of MMRd cells is heavily influenced by the sequence specificity of polymerase proofreading and/or by the copying or incorporation of damaged nucleotides. Mispairing at damaged bases may also contribute to MMRd mutagenesis. Reactive oxygen species (ROS) arising from cellular metabolism can damage genomic DNA; the major mutagenic base damage is the formation of 8-oxo-2′-deoxyguanosine (8-oxoG) (20). 8-oxoG can base pair with adenine, resulting in C > A transversion mutations in the newly synthesised strand (21). Given that MutSα can bind to and initiate the repair of base pairs that contain 8-oxoG (22,23), 8-oxoG formation may be at least partially responsible for the elevated C > A mutagenesis in MMRd cells (24). However, the global contribution of ROS to mismatch formation is not clear.

Cell lines and organoids with MMR gene mutations have been used to confirm and dissect the mutagenic processes observed in cancer (12,24–26). There is a remarkable agreement between the mutation spectra of MutSα deficient (*MSH2* or *MSH6* mutant) cell lines from different species, which all show a higher contribution of the CpG-independent MMRd-B spectrum than typical tumour genomes (12). To better understand the role of oxygen and ROS in mutagenesis, we surveyed the spontaneous mutagenic processes of cultured human cells exposed to different oxygen concentrations. We found a strong dependence of the level of spontaneous mutagenesis on the level of atmospheric oxygen in both MMR proficient and deficient cell lines, indicating a significant role of ROS in spontaneous base substitution mutagenesis. A detailed analysis of the spectrum, strand bias and replication timing distribution of mutations suggested that the oxygen-induced mutations primarily arise from polymerase errors rather than as a consequence of oxygen-induced base damage. Low and high oxygen–specific mutations were detectable in MMRd tumour genomes, and their contribution to subclonal mutation sets may provide information about the hypoxic status of cancer cells.

## Materials and methods

### Reagents

Chemicals used in this study: bromophenol blue (Merck, Darmstadt, Germany, #114391), CMH-HCl (Enzo Life Sciences Inc., Farmingdale, NY, USA, #ALX-430–117-M010), DTPA (Merck, Darmstadt, Germany, #32319), DTT (Merck, Darmstadt, Germany, #D9779), glycerol (Merck, Darmstadt, Germany, #G7757), KCl (VWR, Radnor, PA, USA, #26759), NaCl (Lach-Ner s.r.o., Czech Republic, Neratovice, #30093-AP0), KH_2_PO_4_ (Reanal Zrt., Budapest, Hungary, #17891–1-01–38), methanol (Merck, Darmstadt, Germany, #34860), Na_2_HPO_4_ (VWR, Radnor, PA, USA, #28040), rotenone (Merck, Darmstadt, Germany, #557368), SDS (Serva, Heidelberg, Germany, #20760), TRIS base (VWR, Radnor, PA, USA, #103157P), Tween-20 (Merck, Darmstadt, Germany, #P9416).

Cell culture reagents used in this study: RPMI-1640 medium (Thermo Scientific, Gibco, Waltham, MA, USA, #21875-034), McCoy's 5A medium (Thermo Scientific, Gibco, Waltham, MA, USA, # 16600082), foetal bovine serum (Thermo Scientific, Gibco, Waltham, MA, USA, #10270-106), penicillin-streptomycin solution (Lonza, Basel, Switzerland, #DE17-602E).

Antibodies used in this study: anti-actin (Merck, Darmstadt, Germany, #A5060), anti-tubulin (Merck, Darmstadt, Germany, #T6199), anti-HIF-1 alpha (Abcam, Cambridge, UK, #ab179483), anti-NQO1 (Santa Cruz, Dallas, TX, USA, #sc-32793), anti-HSP10 (Santa Cruz, Dallas, TX, USA, #sc-376313), anti γH2AX JBW301 (Merck, Darmstadt, Germany, #05–636), anti-rabbit IgG-HRP (Merck, Darmstadt, Germany, #A0545), anti-mouse IgG-HRP (Merck, Darmstadt, Germany, #A9044)

### Biological resources

Cell lines used in this study: DLD-1 (ATCC, Manassas, VA, USA, #CCL-221), HCT116 ECACC, Salisbury, UK, #91091005), HCT116 + chr3 (a kind gift from C. Richard Boland (Baylor University, Dallas, Texas, US)) (27), TK6 (JCRB, Ibaraki, Japan, #JCRB1435).

### Cell lines and cell culture

The DLD-1 and TK6 cell lines were cultured in RPMI-1640 basal medium, HCT116 and HTC116 + chr3 cell lines were cultured in McCoy's 5A basal medium, both complemented with 10% foetal bovine serum and 1% penicillin–streptomycin in a humidified incubator with 5% CO_2_ at 37°C. A single cell clone of each cell line was isolated and grown for 60 days either in 3% O_2_ (low oxygen condition) or in standard maintenance conditions with atmospheric oxygen injection that results in 18.6% O_2_ (27) (high oxygen condition, rounded to 19% O_2_ in the text) in several independent replicates. After 60 days a single cell clone was isolated from each replicate and was propagated for DNA isolation. The DLD-1 high oxygen samples were also included in a previous publication (28). For the generation of the DLD-1 *REV1^–/–^* cell line, oligonucleotides encoding a guide RNA targeting the sequence GAAGGGCAGCAAATACCTCAGGG were cloned into the pSpCas9(BB)-2A-GFP vector (Addgene #48138), and cells were transfected with the CRISPR construct using a 4D Nucleofector with P3 Primary Cell transfection reagent (Lonza). 24 hours following transfection, GFP + cells were selected, and single cells were seeded using a BD FACSAria™ III High Sensitivity Flow Cytometer. Clones were grown for approximately 3 weeks, followed by genomic DNA isolation and amplification of the CRISPR target region using Phire Tissue Direct PCR Master Mix (Thermo Fisher Scientific) and primers TGGTCATGTGATAGTGGCTGG and GCTCTTAATGCAACAGCTTAGACT. Mutant clones were identified using T7 Endonuclease I assays (New England Biolabs). Biallelic frameshift mutations altering the sequence at the start of exon 20 from CAAATACCTC to CAAAATTC and CAATTC were confirmed by Sanger sequencing (Microsynth GmbH).

### Immunoblotting

Cultures of the adherent DLD-1, HCT116 and HCT116 + chr3 cell lines were maintained in the appropriate oxygen concentration in 6 cm diameter dishes for 72 h. Media from 80% confluency adherent cultures were removed quickly and completely on ice, then cells were scraped in 4× Laemmli buffer (200 mM Tris (pH 6.8), 400 mM DTT, 8% SDS, 40% glycerol, 0.08% (w/v) bromophenol blue), and boiled immediately for 5 min to preserve HIF-1α integrity. TK6 suspension cell cultures were placed on ice in the flasks; the filter cap was sealed with parafilm immediately. Cooled cultures were centrifuged and cell pellets were boiled in 1 volume of 4× Laemmli buffer. Samples were resolved on appropriate concentration SDS-PAGE. Semi-dry transfer was performed onto 0.2 μM pore size PVDF membrane (Bio-Rad, Hercules, CA, USA, #1620177), then blocked in 5% non-fat milk in TBST (20 mM TRIS pH 7.6 and 150 mM NaCl, 0.1% Tween 20). Primary antibodies were applied in 1:1000 dilution for 1 h in 2.5% non-fat milk in TBST and after washing, membranes were probed with appropriate secondary antibodies. Clarity Western ECL Substrate (Bio-Rad, Hercules, CA, USA, #102031863) was applied to the membranes and chemiluminescent signal was detected by a ChemiDoc MP Imager (Bio-Rad, Hercules, CA, USA) and analysed using the ImageLab software (Bio-Rad, Hercules, CA, USA). Each experiment was repeated independently at least three times.

### Electron paramagnetic resonance spectroscopy

Cells were seeded into 6 cm diameter dishes for 6 hours, then the cultures were grown in low or high oxygen for three days. 5 μM rotenone was added 2 h before sample preparation for electron chain uncoupling. PBS-DTPA (137 mM NaCl, 270 μM KCl, 10 mM Na_2_HPO_4_, 180 μM KH_2_PO_4_ and 1 mM DTPA) was equilibrated with the corresponding oxygen level in the cell culture chambers overnight. CMH-hydrochloride nitrone spin probe was dissolved in ice cold 3% O_2_ equilibrated PBS-DTPA to a 2 mM working solution. Sample preparation from 3% O_2_ cultures were performed in a N_2_ gas filled Atmosbag (Merck, Darmstadt, Germany, #Z530212) in a cold room at 4°C. High oxygen samples were prepared in open air at 4°C. Cultures were washed with the corresponding oxygen equilibrated PBS-DTPA then 1 ml CMH-PBS-DTPA was added to the cultures and cells were incubated with the spin probe for 20 min on a shaker. After incubation, cells were scraped, suspended, and 200 μl of the suspension was snap frozen in liquid nitrogen in 5/4 mm outer diameter/inner diameter defect-free quartz ESR tubes (Wilmad-LabGlass, Vineland, NJ, USA, #701-PQ-7), and stored at −80°C until measurement.

EPR measurements were carried out on a commercial Bruker Elexsys E500 X-band (0.34 T, ∼9.4 GHz) spectrometer equipped with the standard Bruker SHQE cavity (Bruker, Billerica, MA, USA). A temperature of -100°C was reached in the cavity using a custom-built liquid nitrogen flow cryostat (the temperature was stabilized using an Oxford ITC502 temperature controller). The thermocouple was placed in the flow, close to the edge of the cavity, but without perturbing its Q factor. A continuous flow of dry nitrogen gas (purge) was applied to avoid moisture and droplet formation on the walls of the cavity. Special care was taken to employ a low microwave excitation power (0.2 mW) with low magnetic field (4 G) modulation to avoid any saturation and distortion to the ESR line shapes.

The obtained EPR spectra were analysed using the standard double routine (29). The observed EPR line was windowed to remove residual noise. Before each integration step care was taken to remove the linear background. We compared our results by fitting the central line with a derivative Gaussian line, a common practice for spin packets (30). Apart from a global constant scaling factor, we obtained the same results. Data were normalised to the corresponding untreated high oxygen samples for each biological replicate.

### dNTP pool measurement

Approximately 20 million cells of 90% confluent cultures of DLD-1, HCT116 and HCT116 + chr3 that were maintained for three days in low and high oxygen were harvested and washed twice with PBS. Cells were counted using a Vi-CELL XR Viability analyser (Beckman Coulter, Indianapolis, IN, USA). Cell pellets were suspended in 600 μl 60% methanol and kept at −20°C for at least one night. The dNTP pool was extracted at 95°C for 5 min, then samples were centrifuged, the dNTP containing supernatant was dried under vacuum at 45°C (Eppendorf, Hamburg, Germany) and dissolved in 50 μl water. dNTP quantification was performed according to Purhonen et al. (31). Briefly, a 40-fold sample dilution was found optimal for dNTP incorporation assays regarding both the dynamic range of amplification and inhibition of the PCR reaction by the sample. To determine the concentration of a specific dNTP, a qPCR reaction was performed with 0.25 μM 197 nt long template oligo specific to the dNTP to be measured (IDT, Coralville, Iowa, USA), 0.275 μM primer (Merck, Darmstadt, Germany), 1x Q5 polymerase buffer (New England Biolabs, Ipswich, MA, USA), 1x EvaGreen (Biotium, San Francisco, CA, USA, #31000), dNTP mix (50 μM for three dNTPs, where the fourth dNTP could only be supplied from the sample), 20 U/10 U Q5 polymerase (New England Biolabs, Ipswich, MA, USA, #M0491) for dATP and dTTP/dCTP and dGTP respectively. Reactions were assembled in a white, 96-well FrameStar plate (Institute of Applied Biotechnologies a.s., Praha-Strašnice, Czech Republic, #4ti-0961) with 5 μl sample in 10 μl final volumes and ran on a Bio-Rad CFX96 qPCR instrument (Bio-Rad, Hercules, CA, USA). Cycling conditions were the following: 10s 95°C, 1 s 75°C—first read of baseline fluorescence, then elongation 50 min at 66°C where fluorescence was measured every 5 min to monitor proper reaction kinetics. End point fluorescence was read for 5 s at 75°C. Calibration series for every dNTP were included in all measurements (0, 25, 50, 100, 200, 300, 400, 500, 600, 700, 800, 900 fmol) and used for absolute quantification of a given dNTP after subtracting the initial raw fluorescence from the end point values.

### Whole genome sequencing and mutation calling

DNA was extracted from 1 million cells using the Puregene Cell Kit (Qiagen, Venlo, The Netherlands, #158043). Library preparation and whole genome sequencing were done at Novogene (Beijing, China) using 2 × 150 bp paired-end format sequencing on the Illumina platform with 30x mean coverage. Alignment of the sequencing reads to the reference genome GRCh38/hg38 was performed with the Burrows-Wheeler alignment algorithm (32), followed by post-processing with the IndelRealigner tool of the Genome analysis Toolkit (GATK, version 3.8) (33).

SBS and short indels (<50 bp) were identified using the IsoMut method developed for multiple isogenic samples (34–36). In brief, after applying a base quality filter of 30, data from all samples were compared at each genomic position and filtered using optimized parameters of minimum mutated allele frequency (0.2), minimum coverage of the mutated sample (5) and minimum reference allele frequency of all the other samples (0.93). The final input set for IsoMut is listed in Supplementary Table S1. Thirty-one genomes (Supplementary Table S1) used in this study were analysed in a single IsoMut run, DLD-1 *REV1^–/–^* samples were analysed in a separate IsoMut run together with the above samples. Hits were also filtered using a probability-based quality score calculated from the mutated sample and one other sample with the lowest reference allele frequency (34), which was 3.5 in case of SBS mutations, 2.1 for insertions and 3.1 for deletions. For SBSs the threshold was determined such that the number of mutations in any ancestral sample (false positives) does not exceed 1% of the mutations of any of its descendants. This resulted in a maximum number of 20 SBSs in the ancestral clones. In the case of both insertions and deletions the maximum number of events allowed in the ancestral clones was 5. All detected mutations are listed in Supplementary Table S2.

### Analysis of WGS data

For karyotype analysis the sequence coverage was determined using indexcov (37), with the default resolution of 16 384 bp of the linear bam index. To decrease the variance of sequencing depth the rolling average over 200 data points was also plotted, centred at each position.

SBS triplet spectra were determined for each sample, and spectrum deconstruction was done using the fit_to_signatures_strict module of the R package MutationalPatterns (38) with a max_delta value of strictness 0.004. The reference signature set used is indicated at each deconstruction. The MMRd-substituted COSMIC set contains MMRd-A, SigD, SigHD, SigOX and the COSMIC v3.3.1 set excluding SBS6, SBS15, SBS20, SBS21, SBS26 and SBS44. The generation of new mutation signatures by non-negative matrix factorisation (NMF) was also performed using MutationalPatterns.

For the analysis of sequence context, EDLogo plots were generated with the R package Logolas (39). For background data a set of random positions were selected. The number of random positions was ten times the number of indentified SBSs. To control for local genomic effects, random positions were chosen in the ±1 kb context of each SBS.

Replication timing information was downloaded from the Replication Domain database (40). Two datasets of HCT116 cells (accession numbers: Int90617792 and Int97243322) were averaged. The replication timing score at the position of mutations was determined by linear interpolation between the nearest data points. For bar plots, replication timing scores of the whole reference set were divided into deciles.

For replicational strand bias, OK-seq data of HeLa cells were obtained from reference (41). The reads were aligned to the human reference genome GRCh38 with the Burrows-Wheeler alignment algorithm (32). RFD was computed for each 1 kb window and were overlapped with SNVs from IsoMut output.

The transcriptional strand for each mutated position was determined using the mut_strand function of MutationalPatterns (38).

The density of mutations in genic and intergenic regions were determined based on the overlap of mutation positions with the transcripts annotated in TxDb.Hsapiens.UCSC.hg38.knownGene.

Human cancer data were collected from the International Cancer Genome Consortium Data Portal (42), data release 28. The mutational spectrum was determined for each sample and deconstructed to the COSMIC reference set excluding MMRd related signatures but supplemented with signatures MMRd-A and MMRd-B from reference (12). Samples with over 1000 mutations attributed to MMRd-A + MMRd-B were considered as MMRd samples and were used for further analysis. In every sample, clonal and subclonal mutations were separated based on the histogram of variant allele frequencies. The border was determined based on a two-center model using k-means clustering. Samples with fewer than 300 mutations in the subclonal or in the clonal category were excluded. All deconstructions were performed with MutationalPatterns (38). Further WES data was obtained from (43) and analysed as the PCAWG data.

### Statistical analyses

Unpaired two-sided *t*-tests were used for comparisons between genotypes, with the number and independence of samples indicated in the figure legends. The contribution of mutation signatures to the spectrum of clonal versus subclonal mutations was compared using paired two-sided *t*-tests. The difference from an expected distribution of mutation numbers, such as replication or transcription strand bias, was assessed using χ^2^ tests on mutation numbers aggregated by genotype. Precise *p* values are reported throughout the manuscript; all source data used for statistical calculations is provided in Supplementary File 1.

## Results

### Establishment of low oxygen, low ROS culture conditions

**Figure 1.**
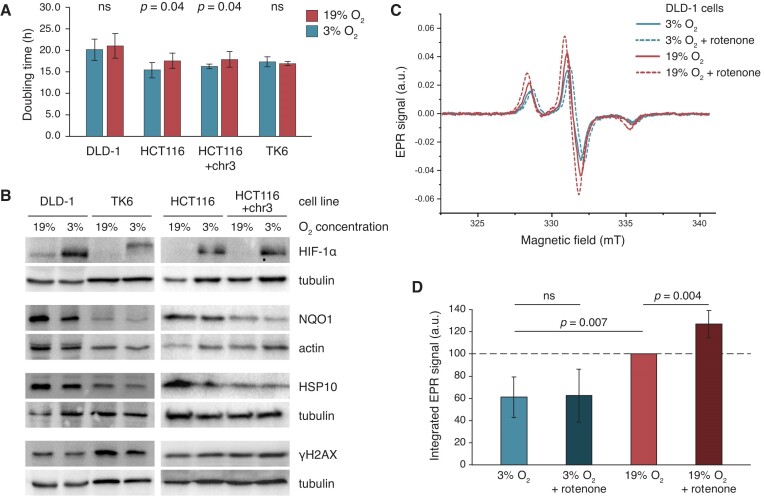
Characterisation of low oxygen culture conditions. **(A)** Doubling time of the investigated cell line cultures kept under different oxygen concentrations (*n* = 6 independent measurements except *n* = 4 for TK6). **(B)** Western blots of whole cell extracts of cells kept at the indicated oxygen concentration for three days. Actin or tubulin was used for loading controls as indicated. Post-translational modifications of HIF-1α (83) are detectable in the TK6 cells. Quantification of the blots is provided in Supplementary File 1. **(C)** EPR spectra recorded from DLD-1 cells kept at the indicated oxygen concentration with or without rotenone (5 μM for 2 h) and incubated with the spin probe CMH for 20 min before collection. Data from a single representative experiment are shown. **(D)** Double integrated EPR signal from three independent experiments as shown in (C), normalised to the high oxygen rotenone-free sample. Mean and SD are shown in (A) and (D), statistical differences are indicated (ns not significant, unpaired two-sided *t*-test).

MMR deficient *MSH6* mutant DLD-1 human colorectal cancer cells with a high spontaneous mutation rate and a typical MMRd mutation spectrum (12,28) and MMR proficient TK6 human lymphoblastoma cells with a low mutation rate (44) were chosen for the study. A second MMRd cell line, the *MLH1* mutant HCT116 colorectal cell line was also included together with its isogenic complemented MMR proficient version in which an extra copy of chromosome 3 provides an intact *MLH1* gene (45). Cells were cultured under atmospheric oxygen injection (approx. 19% O_2_ exposure (27)) or at 3% O_2_, which marginally reduced the growth rate of the HCT116 cell lines (Figure 1A). 3% O_2_ is the approximate lower limit of physoxia in normal tissues, and is higher than oxygen concentrations measured in solid tumours (46). Culturing all cell types for 3 days at 3% O_2_ stabilised HIF-1α, suggesting a chronic hypoxic response (Figure 1B) (47). Hypoxia can induce oxidative stress and ROS production (48,49); cancer cells often respond to hypoxic conditions by upregulating the oxidoreductase enzyme NQO1, whose expression is induced by oxidative stress (50), and by elevating the expression of the chaperone HSP10, which protects unfolded proteins in mitochondria (51,52). However, we did not observe an increased level of NQO1 or HSP10 when cells were kept in 3% O_2_ (Figure 1B, Supplementary File 1). Phosphorylation of histone H2AX, a marker of DNA breaks and single stranded DNA, was also not elevated in 3% O_2_, inferring the lack of hypoxia-induced oxidative DNA damage. To confirm that 3% O_2_ did not cause oxidative stress and to compare the levels of ROS under different oxygen concentrations, we directly measured free radicals in DLD-1 cells using electron paramagnetic resonance (EPR) spectroscopy. Using the spin probe CMH, we found that the level of radicals and other one-electron oxidants (53) was significantly elevated in 19% O_2_ as opposed to 3% O_2_ (Figure 1C, D). Inhibition of mitochondrial electron transport with rotenone further increased the level of intracellular radicals in 19% O_2_, but not in 3% O_2_. Rotenone may not cause a detectable increase of ROS in low oxygen because cells or mitochondria incubated under low oxygen concentrations for prolonged periods exhibit a suppression of aerobic respiration through a modulation of cytochrome C oxidase function by molecular oxygen (54,55). Taken together, the experimental setup provides a baseline low oxygen condition with low ROS levels, and an atmospheric high oxygen condition with elevated ROS levels.

### High oxygen elevates MMRd-specific spontaneous mutagenesis

**Figure 2.**
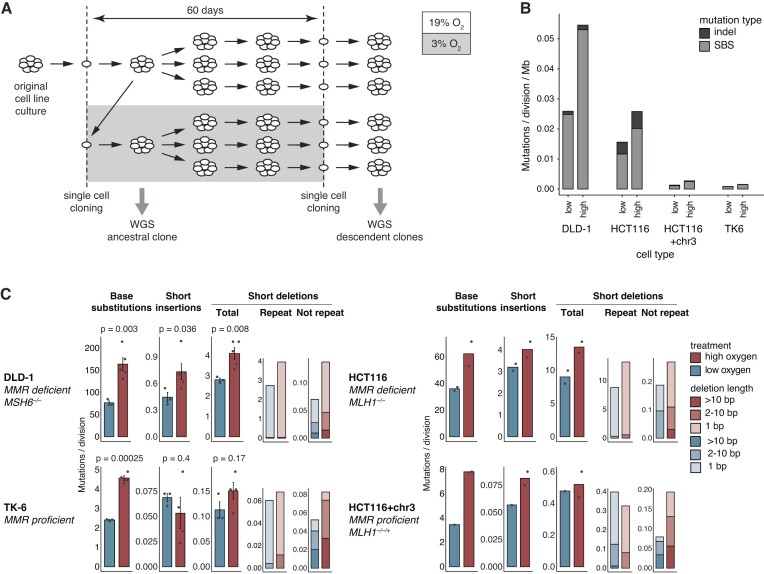
Oxygen-dependent spontaneous mutagenesis in MMR deficient and proficient cells. **(A)** Schematic outline of the long-term cell culture experiments that provided cell clones for whole genome sequencing (WGS). **(B)** The mean rate per megabase of spontaneously arising SBS and short insertion/deletion (indel) mutations in sequenced genomes of the indicated cell types after 60 days of culturing in high or low oxygen conditions. Mutation rates were calculated by dividing the totals with the number of population doublings calculated from the mean population doubling times. **(C)** Mean mutation numbers per population doubling shown separately by mutation class, individual values are also shown. In case of DLD-1 and TK6 cells (*n* = 3 to *n* = 5) error bars indicate SEM of the column totals and statistical differences are indicated (unpaired two-sided *t*-test). A sub-classification of deletion events at repeats or not at repeats is shown in the rightmost two column charts, shaded according to size. The MMR proficiency status and relevant MMR genotype of each cell line is shown.

To assess the effect of oxygen on spontaneous mutagenesis, isogenic cells isolated from a single DLD-1, TK6, HCT116 or HCT116 + chr3 clone were kept in parallel cultures at high (19%) or low (3%) oxygen conditions (Figure 2A). Sixty days after the initial cloning step a single clone was isolated from each culture. Genomic DNA from the ancestral and descendent clones was sequenced, and newly arising mutations were detected using IsoMut, a mutation detection tool designed to identify base substitution and short indel mutations that are unique to a single sample within an isogenic set of samples, thereby efficiently and verifiably filtering out all pre-existing mutations (34). All sequenced cell lines had normal or near-normal, stable karyotypes (Supplementary Figure S1), which facilitated mutation detection. Mutation numbers were normalised to population doublings. As reported (28,44), at high oxygen the spontaneous mutation rate of DLD-1 cells was much higher than that of TK6 cells in case of SBS and short indel mutations (Figure 2B, C). Similarly, the SBS and indel mutation rate of the MMRd HCT116 cells was an order of magnitude higher than that of the chromosome 3 complemented isogenic control (Figure 2B, C). One HCT116 + chr3 descendent clone derived from the low oxygen treatment had lost the extra copy of chromosome 3 (Supplementary Fig. S1) and consequently regained an MMRd phenotype with a high mutation rate. This clone was excluded from all further analyses. In case of the MMR proficient TK6 cells, the SBS numbers were significantly lower in low oxygen, in agreement with reports on human embryonic and adult stem cells (56,57). Importantly, we also observed a significant, more than two-fold difference in SBS numbers in DLD-1 cells kept in low or high oxygen (76.8 ± 7.2 versus 163.8 ± 32.7, *P* = 0.003, unpaired two-sided *t*-test), mirrored in the number of insertions (0.45 ± 0.09 versus 0.73 ± 0.20, *P* = 0.036) and deletions (2.81 ± 0.15 versus 4.10 ± 0.63, *P* = 0.008) (Figure 2C). The analysis of HCT116 cell clone genomes confirmed our findings that MMRd-associated mutagenesis is strongly oxygen-dependent (Figure 2C). The oxygen-dependence of deletions in both DLD-1 and HCT116 cells was largely due to deletions at repeats (Figure 2C), which make up the majority of deletions in MMRd cells and represent the MSI phenotype. The higher number of deletions in HCT116 cells is presumably due to the *MLH1* mutation in these cells inactivating MutLα, which is necessary for the MutSβ-mediated processing of insertion-deletion loops (8). Our observations suggest that protection from oxygen-induced base substitutions and indels is a major role of the MMR pathway.

### Oxygen-induced base substitutions are dominated by T > C transitions in MMRd cells

**Figure 3.**
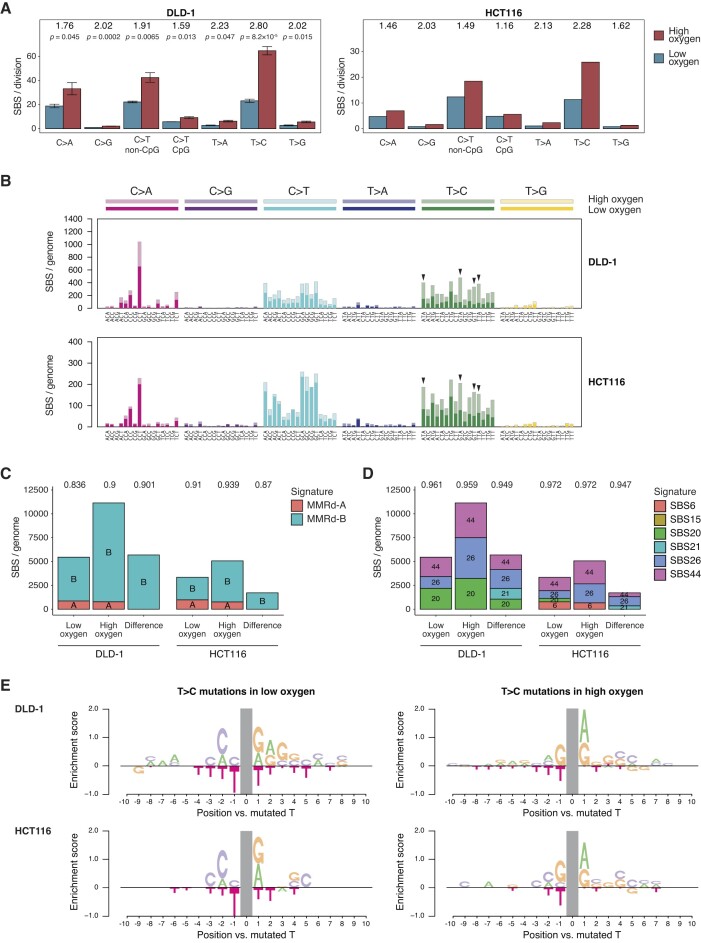
Analysis of the oxygen-dependent MMRd SBS spectrum. **(A)** Spontaneously arising base substitutions by type in sequenced genomes of DLD-1 and HCT116 cells. C > T changes at CpG sequences and other C sites are shown separately. The ratio of high to low oxygen measurements is shown above the columns. Mean and SEM are shown for DLD-1, statistical significances are indicated (unpaired two-sided *t*-test without multiple comparison correction). The HCT116 data are the mean of two measurements. **(B)** The mean spontaneous triplet SBS spectrum of DLD-1 and HCT116 cells kept in high oxygen (pale colours) or low oxygen (saturated colours). Base changes, shown above the panel, are split into 16 categories based on the identity of the preceding and following bases, shown below. (C, D) Deconstruction of DLD-1 and HCT116 spontaneous SBS spectra measured in high or low oxygen, and of the difference of the two spectra, into the previously published signatures MMRd-A and MMRd-B **(C)** and into MMRd-associated COSMIC v3.2 SBS signatures **(D)**. Cosine similarities of the original and reconstructed spectra are shown above the columns. **(E)** Base preferences in the 10-nucleotide context of spontaneous T > C mutations observed in DLD-1 and HCT116 cells kept in high or low oxygen.

Oxidative base damage has been suggested to contribute to mutagenesis in both MMR proficient and MMRd cells (58,59). To better understand the effect of oxygen, we compared the base substitution spectra obtained under low or high oxygen concentration. All base substitution types were more frequent in high oxygen in the MMR proficient TK6 and HCT116 + chr3 cells with the exception of C > T transitions at CpG sequences, but the greatest and most significant differences were seen in case of C > A, C > G and C > T mutations (Supplementary Fig. S2A). All SBS types were significantly induced by high oxygen in the MMRd DLD-1 and HCT116 cells, but the greatest oxygen-dependent increase (both numerically and proportionally) was seen with T > C mutations, which were 2.80 or 2.28 times more frequent in high oxygen, respectively (Figure 3A). When viewed in the context of neighbouring bases, it is apparent that oxygen-induced T > C mutagenesis is highly dependent on the sequence context, with the greatest effect seen at ATA, GTA, GTT and TTA triplets in both cell lines (Figure 3B). Oxidative base damage primarily affects guanine, the base with the lowest oxidation potential (60), but the dominance of T > C substitutions amongst oxygen-induced SBS mutations argues against guanine damage as the major oxygen-dependent mutagenic impact in MMRd cells.

It is important to note the difference between oxygen-induced SBS mutagenesis in MMR proficient and deficient cells. Oxygen induces all SBS types in all cell types, but to different degrees (Supplementary Figure S2A), giving rise to different spectra, and the triplet context of the most common base substitution types is also different in MMR proficient and deficient cells (Supplementary Figure S3A). Also, oxygen induces one or two orders of magnitude more mutations in MMRd cells. Taken together, it appears that we can observe two different oxygen-induced mutagenic processes instead of the lack of MMR simply multiplying the oxidative effect seen in MMR proficient cells: a moderately mutagenic process inducing mainly C > A and C > T is observable in MMR proficient cells, and a highly mutagenic process inducing mainly T > C changes observable in MMR deficient cells.

The deconstruction of the obtained MMRd spectra to the previously defined major components of cancer-specific MMRd mutagenesis (12) showed that oxygen affects the contribution of signature MMRd-B, which is dominated by T > C mutations (Figure 3C, Supplementary Figure S2B, C). Indeed, the difference between high and low oxygen conditions is reasonably well approximated by MMRd-B alone (cosine similarity 0.901 and 0.870 for DLD-1 and HCT116 cells, respectively, Figure 3C). Deconstruction to MMRd-associated COSMIC SBS signatures shows that the contributions of the T > C dominated SBS26 as well as the common SBS44 are most affected by oxygen (Figure 3D, Supplementary Fig. S2C). The spectrum of the oxygen-induced base substitutions revealed by the difference of high and low oxygen spectra showed a unique contribution by SBS21. Concordantly, the difference spectrum was most similar to MMRd-B or SBS21 and SBS26 (see Supplementary Figure S3C, which also shows further comparisons to MMRd-associated cancer mutation signatures). The highest T > C peaks are identical in SBS21 and in the oxygen-induced difference spectrum (Supplementary Figure S3C), suggesting that SBS21 comes close to capturing oxygen-induced mutagenesis in MMRd cells.

An analysis of the wider sequence context of T > C mutations revealed oxygen-dependent but cell line–independent differences (Figure 3E). In low oxygen there is a general negative preference for thymines in the vicinity, and a strong enrichment revealing positive preference for C at the −2 position. In contrast, in high oxygen there is strong preference for G at the −1 position and A or G at the +1 position. The context of C > A and C > T mutations did not show such an oxygen dependence (Supplementary Figure S4), supporting the conclusion that a distinct oxygen-dependent T > C dominated mutagenic process can be observed in MMRd cells.

### MMRd mutagenesis is influenced by replication but not transcription

**Figure 4.**
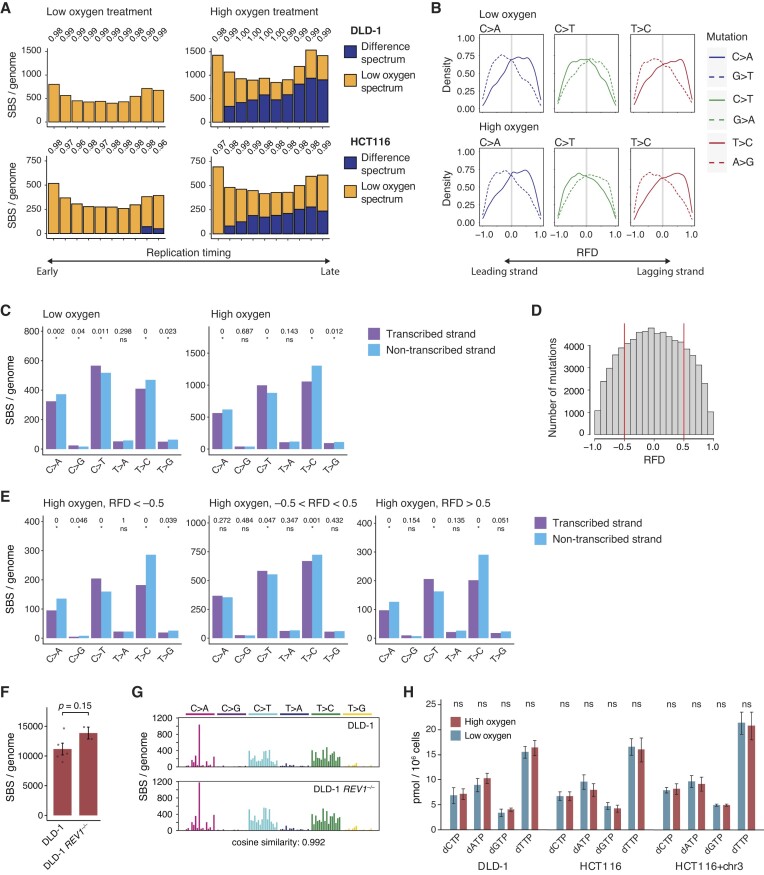
Exploring the mechanism of oxygen-dependent MMRd mutagenesis. **(A)** SBS mutations in MMRd DLD-1 or HTC116 cells kept in low or high oxygen were assigned to equal deciles of the genome defined by replication timing, and each mutation set was deconstructed into a mixture of two components defined as the low oxygen spectrum and the difference of the high and low oxygen spectra of the respective cell line. Cosine similarities of the original and reconstructed spectra are shown above the columns. **(B)** Distribution of the major SBS mutation classes in DLD-1 cells kept in low or high oxygen, relative to replication fork directionality (RFD). Positive RFD indicates a rightward-oriented fork, in which case the sense strand is the lagging strand. RFD is calculated by the difference between rightward- and leftward-oriented forks; thus, a value of 0 means that, at that location, equal numbers of forks go to the right and to the left. **(C)** Strand bias of SBS mutations observed in genic genomic regions of DLD-1 cells kept in low or high oxygen. The statistical significance of the differences is shown above the columns, calculated from the total number of mutations summarised from all relevant samples, * *P* < 0.05, ns not significant, χ^2^ test. **(D)** The number of SBS mutations in genomic regions with different RFD values in DLD-1 cells kept in high oxygen. **(E)** Strand bias of SBS mutations observed in transcribed genomic regions of DLD-1 cells kept in high oxygen, plotted separately for mutations at the indicated RFD value ranges. Statistical significances are shown as in (C). **(F)** Newly arising SBS mutations detected after 60 days of culturing in high oxygen in two DLD-1 *REV1^–/–^* cell clones. The five control clones presented on Figure 2C are shown for comparison. Error bars indicate SEM, statistical significance is shown (unpaired two-sided *t*-test). **(G)** The mean spontaneous triplet SBS spectrum of the indicated cell lines, formatted as in Figure 3B. The similarity of the two spectra is indicated. **(H)** Measurement of dNTP levels in the indicated cell lines following three days at low or high oxygen conditions. *n* = 3 independent experiments, error bars indicate SEM, ns not significant, unpaired two-sided *t*-test.

We next analysed the genome-wide distribution of SBS mutations to ask whether oxygen-induced MMRd mutagenesis arises from oxidative DNA damage or mismatched intact nucleotides. Dividing the genome into equal parts by replication timing, we found a complex distribution of mutation densities including an enrichment of mutations in the earliest replicating decile of the genome of DLD-1 and HTC116 cells kept in high or low oxygen (Figure 4A). When we split the mutation sets into two components defined as (i) the SBS spectrum of the cell line in low oxygen and (ii) the difference of the low and high oxygen specific SBS spectra of the respective cell line (see Figure 3B), we found that these were both present after high oxygen treatment, and were affected by replication timing in opposing manner. The oxygen-stimulated component was replication-dependent and enriched in the late replicating regions, with a gradual increase of mutation density with progressively later timing (Figure 4A), mirroring the replication timing distribution in cancer genomes of the T > C dominated signature SBS21 (61), which we found to be associated with high oxygen environment in cell culture.

Using replication fork direction data derived from Okazaki fragment sequencing (41) we found that the oxygen level did not influence the strand bias of different SBS classes: as reported previously, we observed C > A and T > C bias towards the lagging strand, and C > T bias to the leading strand (equivalent to G > T and A > G leading strand bias, and G > A lagging strand bias, respectively) (Figure 4B, Supplementary Figure S5A). Thus, the oxygen-induced mutations also show strong replication strand bias, indicating their replication-dependent origin.

Transcriptional strand asymmetry has been described for MMRd-associated SBS mutations, particularly an enrichment of T > C mutations on the untranscribed strand in signatures SBS21 and SBS26, suggesting that T > C mutations arise at thymine lesions that are subject to transcription-coupled repair (11). In apparent agreement, we did find a significant enrichment of T > C mutations on the untranscribed strand in high oxygen, as well as significant transcription strand bias of C > A and C > T mutations (Figure 4C, Supplementary Figure S6). However, when we corrected for the preferential co-directionality of replication and transcription (41) by splitting the mutation datasets according to the local replication fork direction (RFD) values calculated from the Okazaki sequencing datasets (Figure 4D), we found that in areas of weak replication direction preference (−0.5 < RFD < 0.5) there was only marginal transcriptional strand bias of the same mutation classes in DLD-1 cells (Figure 4E, compare the middle panel to the left and right panels) and no transcriptional strand bias in HCT116 cells (Supplementary Figure S6B). Mutation densities were very similar in genic and intergenic regions, even in case of the most highly oxygen-dependent T > C mutations at ATA, GTA, GTT ad TTA triplets (Supplementary Figure S5C).

If oxygen-induced mutations were due to DNA lesions, error-prone translesion synthesis (TLS) could be responsible for the observed mutation spectra. To explore this, we disrupted the *REV1* gene in DLD-1 cells. REV1 serves as a recruitment platform for multiple TLS polymerases (62) and we have shown that it is responsible for over 50% of spontaneous SBS mutagenesis, including a component presumably arising from oxidised bases, in MMR proficient human cells (63). Following the same experimental pipeline as for the normal DLD-1 controls, we found that the number of newly arising SBS mutations was not lower in two sequenced DLD-1 *REV1^–/–^* cell clone genomes at high oxygen (Figure 4F). Importantly, the mutation spectrum of the *REV1^–/–^* cells was very similar to that of the control cells grown in high oxygen (Figure 4G, cosine similarity 0.992), and less similar to the control grown in low oxygen (cosine similarity 0.963). Thus, REV1-dependent TLS, which could bypass e. g. oxidative thymine glycol lesions (64), is not responsible for oxygen-induced mutagenesis in MMRd cells. The lack of a transcription-coupled effect and the unchanged mutation spectrum in TLS defective cells argues that oxygen-dependent MMRd mutagenesis is caused by an increase in replicative DNA mismatches rather than oxidative DNA lesions.

The altered spectrum of replication-dependent SBS mutations could be potentially caused by an oxygen-dependent change in deoxyribonucleotide (dNTP) availability. We directly measured dNTP pools in DLD-1, HCT116 and HCT116 + chr3 cells under low and high oxygen culture conditions and found no significant oxygen-induced difference in the concentration of any dNTP in any of the cell lines (Figure 4H), ruling out a role of the dNTP pools. Instead, high oxygen may have a direct effect on replication fidelity, increasing in late replicating regions as ROS levels increase through the S phase of the cell cycle (65).

### High oxygen accelerates MMRd-dependent indel mutagenesis

**Figure 5.**
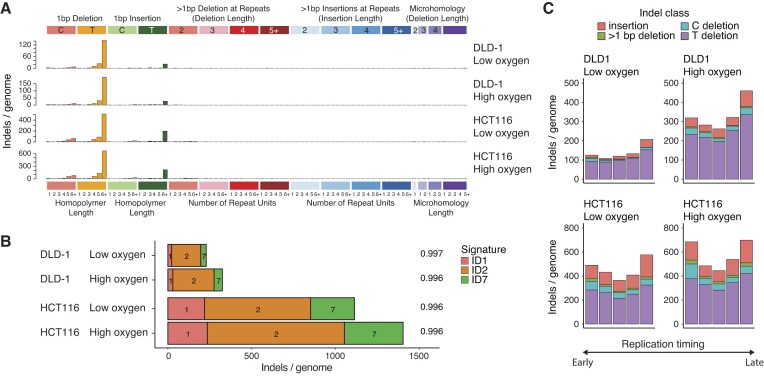
Oxygen-dependent indel mutagenesis. **(A)** Indel spectra of spontaneous mutations detected in DLD-1 and HCT116 cells kept in low or high oxygen for 60 days. **(B)** Deconstruction of the indel spectra shown in (A) into COSMIC indel signatures ID1–ID17. Cosine similarities of the original and reconstructed spectra are shown on the right. **(C)** Indel mutations in DLD-1 or HTC116 cells kept in low or high oxygen were assigned to equal quintiles of the genome defined by replication timing, and each mutation set was categorised according to the key shown.

High oxygen conditions induced significantly more indels in DLD-1 cells and similarly increased the rate of insertion and deletion mutagenesis in HCT116 cells (Figure 2C). The simple, sparse indel spectrum of both MMRd cell lines was dominated by 1 bp deletions and a smaller number of 1 bp T insertions at homopolymeric T repeats (Figure 5A), and did not show any oxygen-dependence. A deconstruction of the spectra to COSMIC indel signatures revealed contributions by the ID2, ID2 and ID7 signatures characteristic of MMRd cancer cells (11), and their relative contributions did not vary with the oxygen level (Figure 5B). Oxygen therefore accelerates MMRd-related indel mutagenesis without changing its spectrum. However, the dominant T deletions showed many parallels with the oxygen-induced T > C mutations including an enrichment in late replicating regions, lagging strand bias, similar rates in transcribed and untranscribed regions, and no transcriptional strand bias (Figure 5C, Supplementary Figure S7), suggesting that they are generated by a related inaccurate replicative process with an oxygen-dependent error rate.

### Oxygen-dependent MMRd mutagenesis changes during tumour evolution and correlates with SBS21

**Figure 6.**
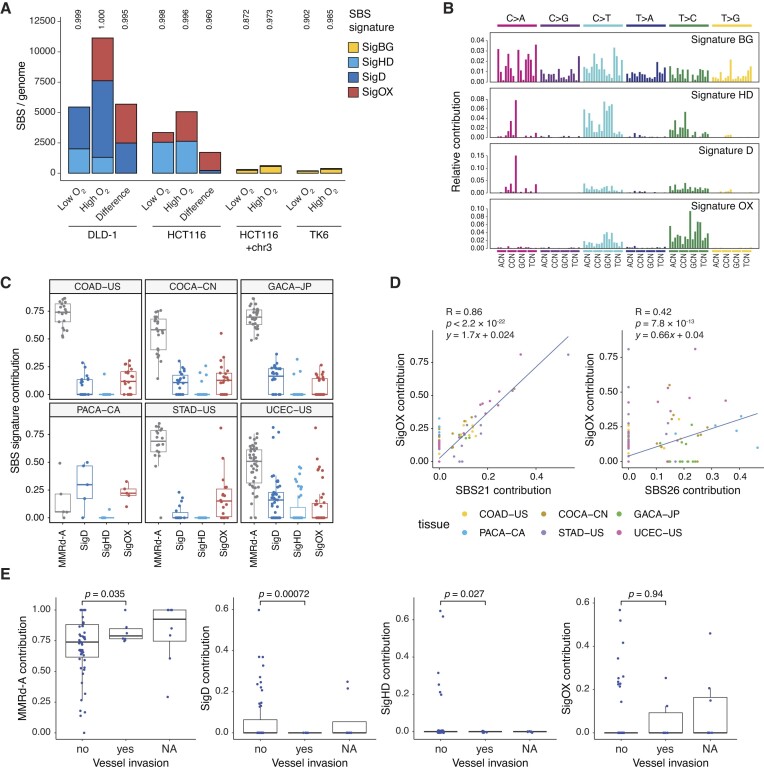
Evidence of oxygen-dependent mutagenesis in MMRd cancers. **(A)** Deconstruction of the triplet SBS mutation spectrum of each cell line and treatment combination and of the difference between the low and high oxygen-specific SBS spectra of MMRd cell lines into the signatures derived from cell line sequencing data by NMF. Cosine similarities of the original and reconstructed spectra are shown above the columns. **(B)** Triplet SBS spectra of MMRd mutation signatures used in (A). Sequence context is shown below the panels; the order of the bases following the mutated position is alphabetical. **(C)** Contribution of the MMRd-A signature and of the three MMRd-associated SBS signatures derived from the cell line samples to the individual mutation sets of MMRd samples of six different PCAWG cohorts. Box plots show the median and the interquartile range. Only tumour samples with positive SigHD + SigD + SigOX contribution are shown. **(D)** Correlations of the indicated signature contributions in deconstructions of MMRd tumour somatic mutation datasets using the entire COSMIC signature set (*x* axis) or the MMRd-substituted set (*y* axis). **(E)** Correlation with blood vessel invasion of the contribution of MMRd-A, SigD, SigHD and SigOX to the subclonal mutations of MMRd colorectal cancer whole exomes deconstructed using the reference signatures MMRd-A, SigD, SigHD, SigOX, SBS1, SBS10a and b. The significance of the differences is indicated (unpaired two-sided *t*-tests).

We next examined the relevance of the experimentally observed effects of oxygen to the biology of MMRd cancers. For this purpose, we sought to identify a unified oxygen-induced mutational process in the two MMRd cell lines used in this study. Using NMF on the triplet mutational catalogues of all sequenced cell clones, we found that four mutational signatures adequately describe mutagenesis in all samples (Figure 6A, B). A broad-spectrum background signature described mutagenesis in the MMR proficient cells (SigBG). A ‘general’ MMRd signature was present in both DLD-1 and HCT116 genomes (SigHD) and did not respond to oxygen treatment, whereas SigD was only present in the DLD-1 mutation set. Most importantly, the fourth signature (SigOX) showed very strong correlation with oxygen exposure in MMRd cells: it was only present in the high oxygen DLD-1 samples, and described the majority of the difference between high and low oxygen conditions in both DLD-1 and HCT116 genomes (Figure 6A). The oxygen-induced SigOX is dominated by T > C mutations and captures the four strongest-responding C > T contexts (see Figure 3B).

We analysed the contributions of oxygen-dependent components to the somatic SBS spectra of MMRd samples of the Pan-Cancer Analysis of Whole Genomes (PCAWG) collection. For this purpose, we deconstructed the mutation sets using COSMIC v3.3.1 signatures, but replaced the seven MMRd-associated signatures with the newly defined SigD, SigHD and SigOX, plus the CG > TG dominated MMRd-A signature that is prevalent in cancer mutation sets but makes small contributions in cell culture (12). SigOX was detectable in the majority of samples (Figure 6C). SigOX showed highest similarity to COSMIC signatures SBS21 and SBS26 (cosine similarity values 0.82 and 0.84, respectively, Supplementary Figure S8A), and its contribution showed correlation with that of both SBS21 and SBS26, though the correlation with SBS21 was much stronger (*R* = 0.86, Figure 6D), supporting the association of SBS21 with the mutagenic effects of oxygen.

Clonal mutations detected in cancer samples arise before or during the early stages of tumour evolution. Subclonal mutations reflect tumour heterogeneity, and are guaranteed to have arisen at a later stage than clonal mutations of the same tissue sample. Indeed, we noticed that the spectrum of clonal and subclonal SBS mutations was different in many MMR deficient PCAWG samples, with altered contributions of the cell culture–derived SigHD, SigD and SigOX (Supplementary Figure S8B–D). To better understand the physiological meaning of these variations, we examined the correlation of oxygen-specific MMRd mutation signatures to clinical attributes of MMRd cancers using a colorectal cancer whole exome sequencing dataset by Zhao *et al.* (43). Uniquely, blood vessel invasion significantly correlated with the contribution of SigD and SigHD to subclonal mutations (Figure 6E, Supplementary Figure S9). The low oxygen–specific SigHD was only observed in samples with no blood vessel invasion, presumably identifying a hypoxic environment in these tumours.

## Discussion

Using controlled cell culture conditions, we have shown a dependence of spontaneous base substitution and indel mutagenesis on oxygen concentration in MMRd cells. Increased mutagenesis in high oxygen correlates with an increased concentration of intracellular oxidants, and shows a specific SBS spectrum most similar to the cancer mutation signatures SBS21 and SBS26. The oxygen-induced mutations are dominated by T > C transitions, display lagging strand bias and late replication timing bias, and show no influence of transcription-coupled repair. The experimentally defined high oxygen-specific mutational signature is detectable in MMRd cancer genomes.

The central question arising from observations of oxygen-induced mutagenesis in MMRd cells is whether mutations are a direct consequence of ROS-induced base damage. Considering damage to genomic DNA, the prevalence of T > C substitutions rules out an involvement of the most common oxidative lesions at guanine, which can nevertheless contribute to the oxygen-induced C > A mutations observed in all investigated cell lines. Oxidative damage can also occur at A–T base pairs (66). Of the most prevalent oxidation products, thymine glycol is not a miscoding lesion, and 8-oxoadenine induces A > C(T > G) mutations (67) that are rare in MMRd cells. Further minor products of base oxidisation could contribute to mutagenesis, but those that stall RNA polymerase II would also provide a substrate for transcription-coupled repair (68). We carefully separated the effects of replication and transcription on mutational strand bias using cell line–specific data on replication direction. The lack of transcriptional strand bias argues against an oxygen-dependent mutagenic mechanism caused by transcription-stalling oxidised bases. Oxidative A/T lesions are also not very common. Ames and co-workers had estimated that human cells repair about 320 of the most common thymine glycols per day (69), although this may be more in high oxygen. Even so, the measured 60 T > C mutations per cell doubling in DLD-1 cells could only be caused by oxidative A/T lesions if their bypass by replicative polymerases or TLS, or repair by base excision repair had very much higher mutation rates than reported in the literature (64,70).

Oxidised bases may also be incorporated into DNA from the nucleotide pool during replication. As long as such bases are correctly removed by base excision repair and/or MMR (23) prior to the next replication cycle, this process is not mutagenic. An increased incorporation of 8-oxoG to bacterial genomes due to MutT disruption does result in T > G mutations (71), but we detected few T > G mutations regardless of the oxygen concentration. Taken together, we find it unlikely that ROS-induced base damage is responsible for the mutagenic effect of high oxygen, though we cannot exclude that it makes a contribution.

Alternatively, the oxidising intracellular environment may directly influence the error profile of the replicative polymerases. The mutation spectrum of human proofreading-proficient replicative polymerases has not been accurately measured due to challenges of low error rates and interference from damaged bases. However, the spectrum of oxygen-induced mutations (SigOX) is only subtly different from T > C and C > T components of the overall MMRd SBS spectra, suggesting that it reflects endogenous mistakes by the polymerase, the spectrum shaped by the specificity of base insertion and of proofreading. How could the oxygen level influence polymerase fidelity? Eukaryotic B family DNA polymerases including the replicative polymerases Pol α, Pol δ and Pol ϵ contain a [4Fe-4S] iron-sulfur cluster coordinated by four cysteines in the C terminus of their catalytic subunit (72–74), and several recent studies reported that the integrity of the Fe-S cluster influences the fidelity of human or yeast Pol δ (75–77). Our EPR measurements with the probe CMH detect one-electron oxidants, and Fe-S clusters are prime electron-transfer groups for one-electron redox processes. One possible mechanism explaining our observations is that the increased level of intracellular oxidants in the high oxygen environment alters the oxidation state of the Fe-S cluster in replicative polymerases and thereby reduce replication fidelity in a strand-specific manner. Pol δ appears to be the main source of A > G/T > C mutations in human MMRd cancer cells (78). Regardless of the exact mechanism influencing polymerase fidelity, increased misinsertions of G opposite T by Pol δ could thus be responsible for oxygen-induced T > C mutations with a lagging strand bias.

The oxygen-specific component of MMRd mutagenesis is also captured by mutation signature analyses of cancer genomes. The low oxygen component SigHD common to both investigated MMRd cell lines is very similar to the ‘generic’ MMRd COSMIC signature SBS44 (11) (Supplementary Figure S8A). The high oxygen component SigOX found here in both *MSH2* and *MLH1* deficient cells is most similar to COSMIC SBS21 and SBS26, but correlates better with the contribution of SBS21. The similar SBS21 and SBS26 are both dominated by T > C mutations, and the oxygen effect may account for their separation. Cancer mutation signatures turned out to be excellent at capturing biological processes, therefore the good match between a cancer signature found by unsupervised mutation analysis and the experimentally determined SigOX strongly supports the *in vivo* relevance of our findings. The inactivation of *PMS2* but not of other MMR genes induces a mutation spectrum dominated by T > C mutations (24), PMS2 therefore appears to play a specific role in the repair of oxygen-induced mismatches. Indeed, T > C mutagenesis in *Pms2* null mouse cells was reduced when antioxidants were added to the medium (79). The dominance of T > C mutations in the mutagenic spectrum of PMS2 deficient cells as opposed to those lacking MSH2, MSH6 or MLH1 warrants further investigation.

A limitation of our study is that in DLD-1 cells Pol δ carries an R689W mutation in the POLD1 subunit, and this variant can cause an increased mutation frequency in both MMR proficient and deficient cell lines (80). The R689W mutation may be responsible for the observed approximately 2-fold higher SBS mutagenesis in DLD-1 cells than in HCT116 cells which possess wild type POLD1. The SBS spectrum of the two MMRd cell lines was also slightly different, with SigD only present in DLD-1 cells. SigD only differs from the low oxygen–specific SigHD by the higher prevalence of C > A mutations, which were also the most common mutation class when POLD1^R689W^ was introduced to HCT116 cells (80), therefore SigD appears to capture the effect of this polymerase mutation. Indeed, SigD is most similar to COSMIC SBS20, which is associated with defects in the Pol δ polymerase domain (81). Importantly, the effect of oxygen was captured by SigOX in both DLD-1 and HCT116 cells, showing that the oxygen-dependent T > C and C > T mutagenesis is robustly identifiable and not affected by POLD1^R689W^.

When considering the use of the experimentally defined signatures for assessing the oxygen exposure of tumours, one must note that MMRd tumour genome SBS mutations are dominated by mutations at methylated cytosines described by the MMRd-A signature, which arise in a largely replication-independent manner (14). The relative contribution of this MMRd-A component was typically lower amongst subclonal mutations in whole genome sequenced datasets of PCAWG, presumably due to the higher contribution of replication-dependent mutagenic processes in the faster proliferating cancer cells (see Supplementary Figure S10 for a model). The balance of oxygen-dependent and independent mutational signatures could provide information on the oxygen supply of the sampled tissue, but deep sequencing of tumour genomes is needed for quality datasets of subclonal mutations that enable the analysis of the contributions of replication-coupled mutational processes in developed tumours. Concordantly, detectable low oxygen–specific MMRd mutagenesis was found to negatively correlate with vascularisation amongst subclonal mutations in a relatively high coverage whole exome sequenced dataset. Solid tumours are well documented to be hypoxic (46,82), and careful analyses of later occurring subclonal high or low oxygen–specific mutational populations could report on their recent oxygen exposure and metabolic state.

The investigation of MMR deficient cells sheds light on the processes that create primary mismatches in the genome. In fast proliferating cells, a large proportion of mismatches are caused by DNA replication. We showed that replicative mismatch generation is strongly dependent on oxygen levels, thus a main function of MMR is the protection of the genome from the mutagenic effect of oxygen exposure.

## Supplementary Material

gkad775_Supplemental_Files

## Data Availability

Source data for all figures including raw gel images and numerical data used to generate graphs are provided in Supplementary File 1. Raw sequence data obtained for this study is available from the European Nucleotide Archive under study accession number PRJEB55394.
