# Supplementary material for: DNA mismatch repair protects the genome from oxygen-induced replicative mutagenesis
Source: Nucleic Acids Res. 2023 Oct 4;51(20):11040–55. doi: 10.1093/nar/gkad775 (PMC10639081; doi:10.1093/nar/gkad775)
Supplement: gkad775_Supplemental_Files [file gkad775_supplemental_files.zip › Supplementary_figs.pdf]

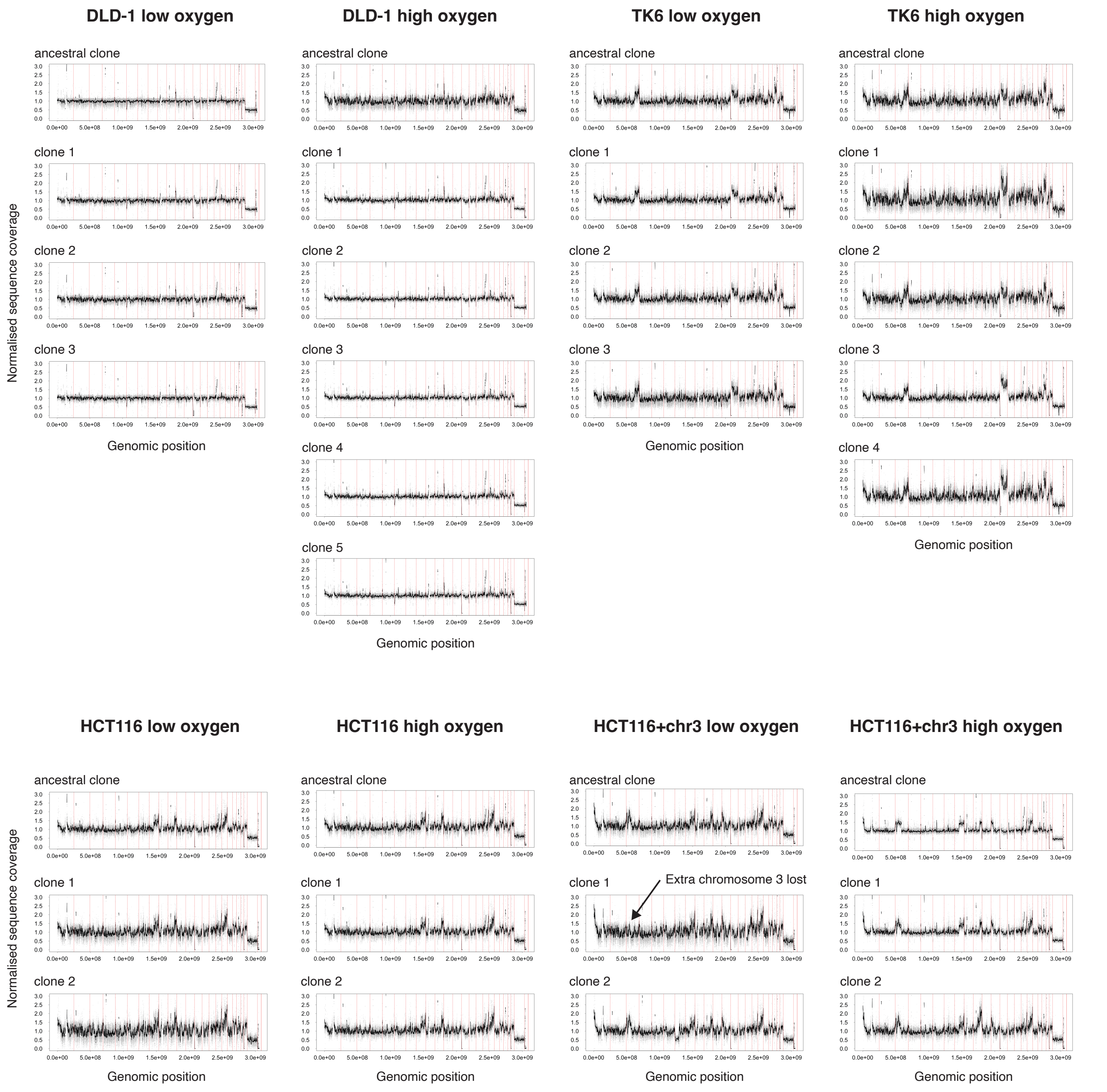

Supplementary Figure S1. Karyotype analysis of the sequenced cell clones.
Sequence coverage of whole genome sequence datasets of analysed cell clones, normalised to the mean coverage of each sample. Chromosome boundaries are indicated with vertical dashed lines. The loss of the extra chromosome 3 fragment in HCT116+chr3 descendent clone 2 is marked.

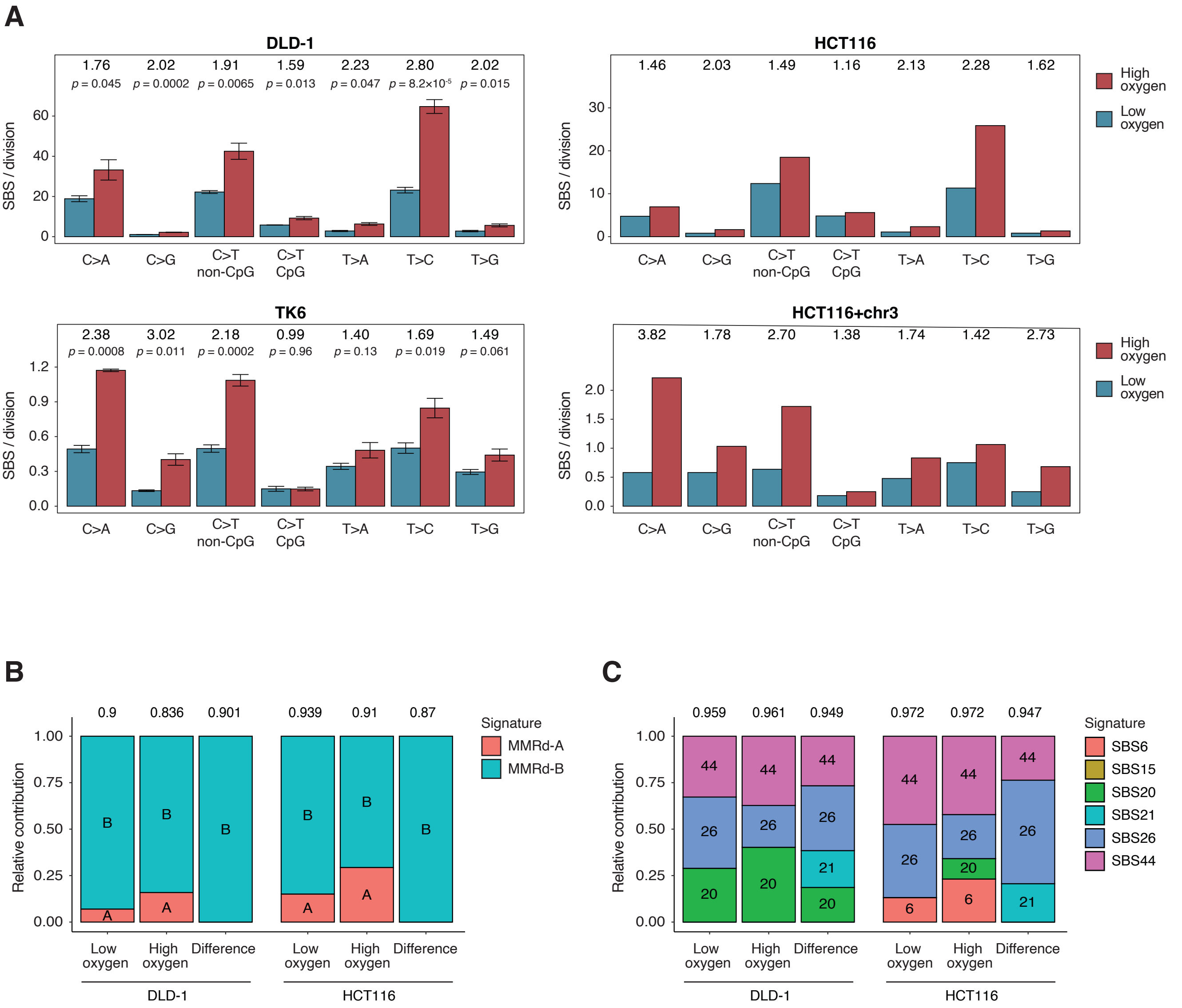

Supplementary Figure S2. Analysis of the oxygen-dependent MMRd SBS spectrum.
(A) Spontaneously arising base substitutions by type in sequenced genomes of the indicated cell types. C>T changes at CpG sequences and other C sites are shown separately. The ratio of high to low oxygen measurements is shown above the columns. Mean and SEM are shown for DLD-1 and TK6 data, statistical significances are indicated (unpaired two-sided *t*-test without multiple comparison correction). (B, C) Deconstruction of DLD-1 and HCT116 spontaneous SBS spectra measured in high or low oxygen, and of the difference of the two spectra, into signatures MMRd-A and MMRd-B (B) and into MMRd-associated COSMIC v3.3.1 SBS signatures (C). Cosine similarities of the original and reconstructed spectra are shown above the columns.

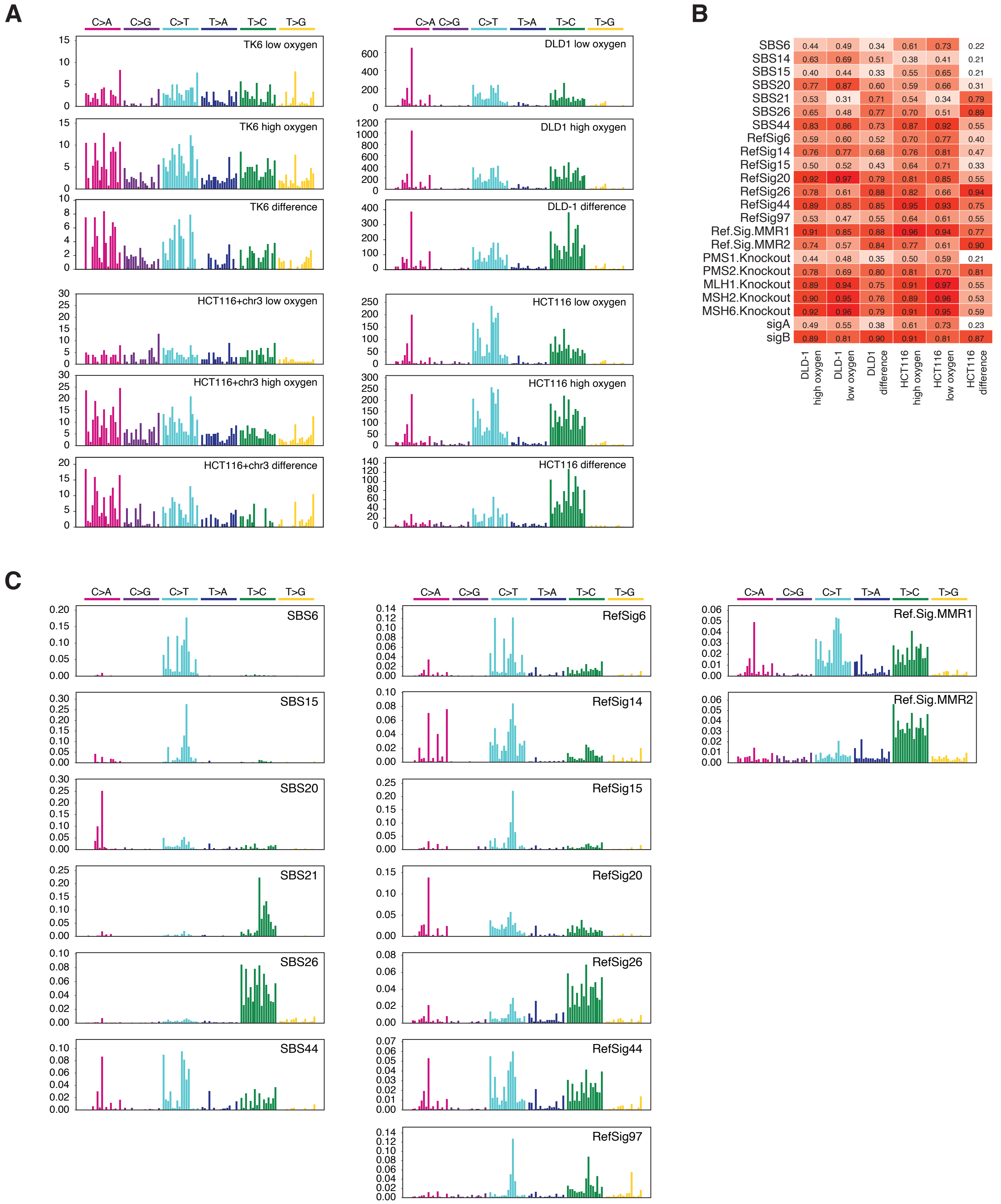

Supplementary Figure S3. SBS triplet mutational spectra and signatures.

(A) Averaged spectra of spontaneously arising mutations in the indicated cell lines kept in low or high oxygen are shown in 96 categories based on the sequence context of mutations. The difference spectra calculated as high oxygen spectrum minus low oxygen spectrum are also shown. Negative peaks due to experimental error in case of low mutation numbers are shown as zero. (B) Cosine similarity of the detected MMRd mutation spectra to mutational signatures described in our previous study (MMRd-A and MMRd-B, [Németh E et al., DNA Repair 89:102827. (2020)]) and published in literature. SBS6-SBS44 are reference signatures from COSMIC [Alexandrov LB et al., Nature, 578, 94-101. (2020)], RefSig6-RefSig97 are from [Degasperi A et al., Science, 376, 6591 (2022)], Ref.Sig.MMR1 and 2 are from [Degasperi A et al., Nat Cancer, 1(2):249-263. (2020)]. Mutational spectra of MSH2, MSH6, MLH1, PMS1 and PMS2 knockout cell lines are from [Zou X et al., Nat Cancer, 2(6), 643-657. (2021)]. (C) MMRd-related reference SBS mutational signatures as used in (B).

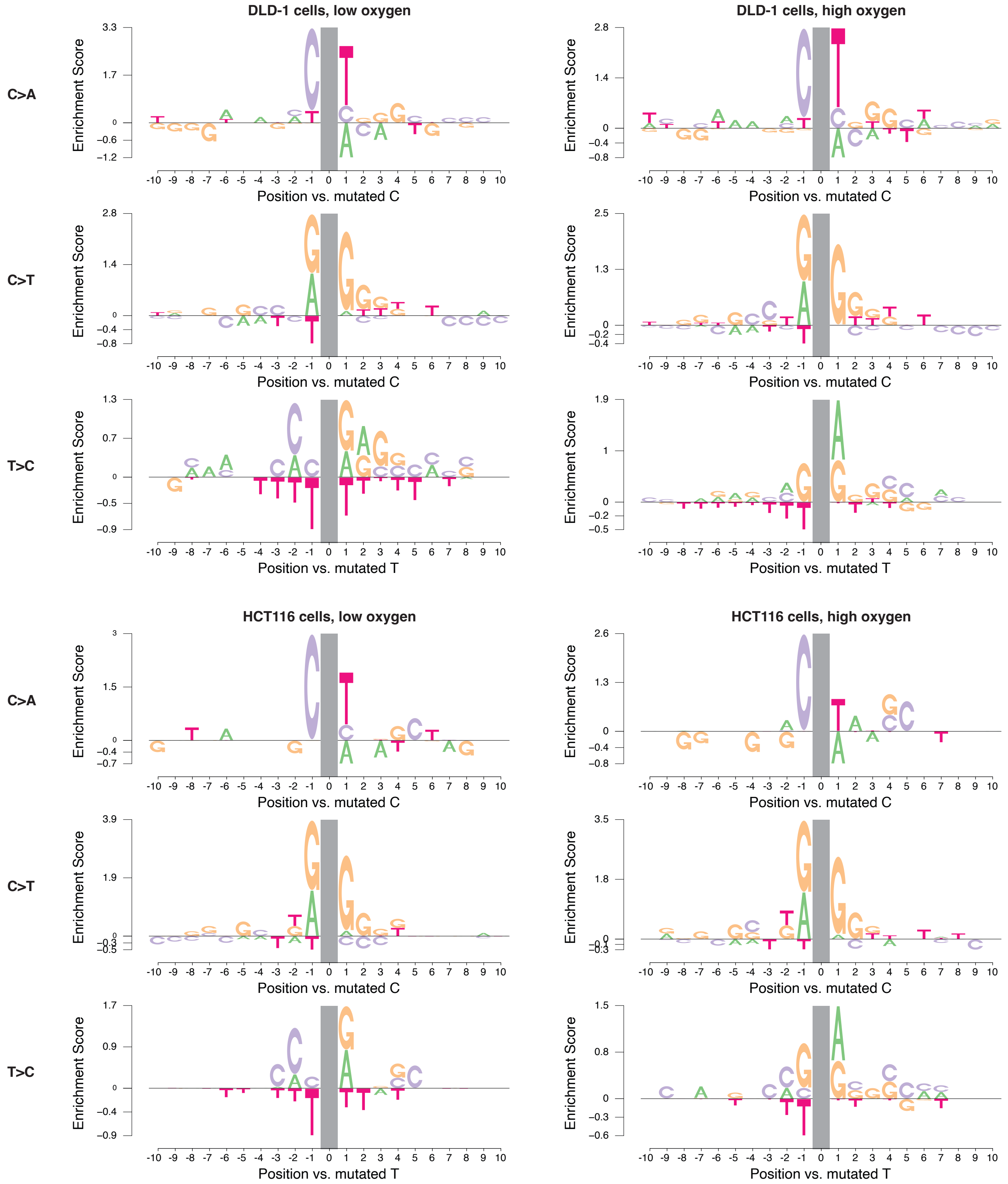

Supplementary Figure S4.

Base preferences in the 10-nucleotide context of spontaneous C>A, C>T and T>C mutations observed in DLD-1 and HCT116 cells kept in low or high oxygen as labelled.

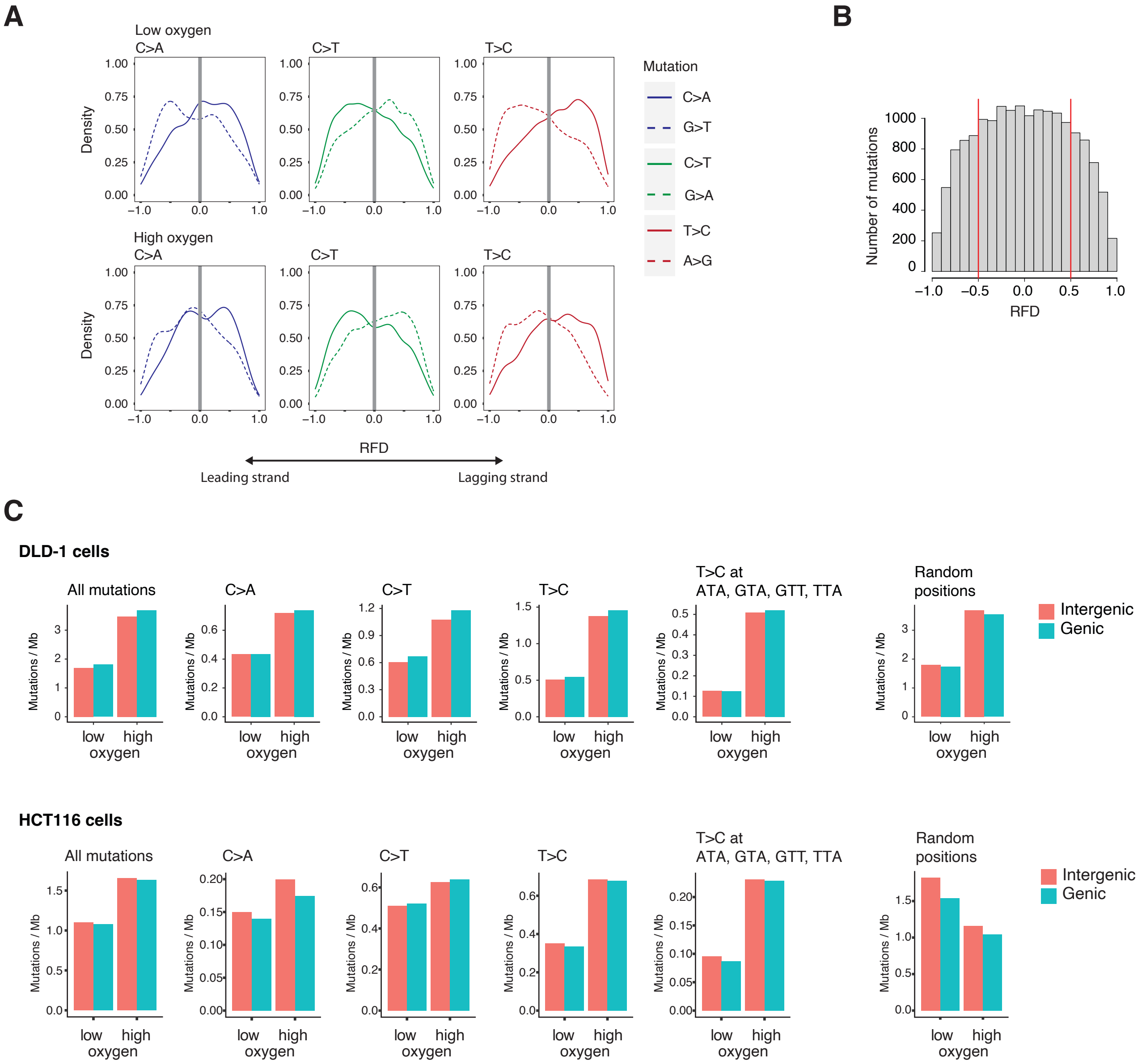

Supplementary Figure S5. Correlation of SBS mutagenesis with genomic features.
(A) Distribution of the major SBS mutation classes relative to replication fork directionality (RFD) in HCT116 cells kept in low or high oxygen. Positive RFD indicates a rightward-oriented fork, in which case the sense strand is the lagging strand. RFD is calculated by the difference between rightward- and leftward-oriented forks; thus, a value of zero means that, at that location, equal numbers of forks go to the right and to the left. (B) The number of SBS mutations in genomic regions with different RFD values in HCT116 cells kept in high oxygen. (C) Mutation densities in genic versus intergenic regions in DLD-1 cells (top row) and HCT116 cells (bottom row), shown separately for all SBS mutations and for mutations of the indicated sequence category. Mutation densities for the equal number of spectrum-matched randomly generated control positions are shown on the last panel.

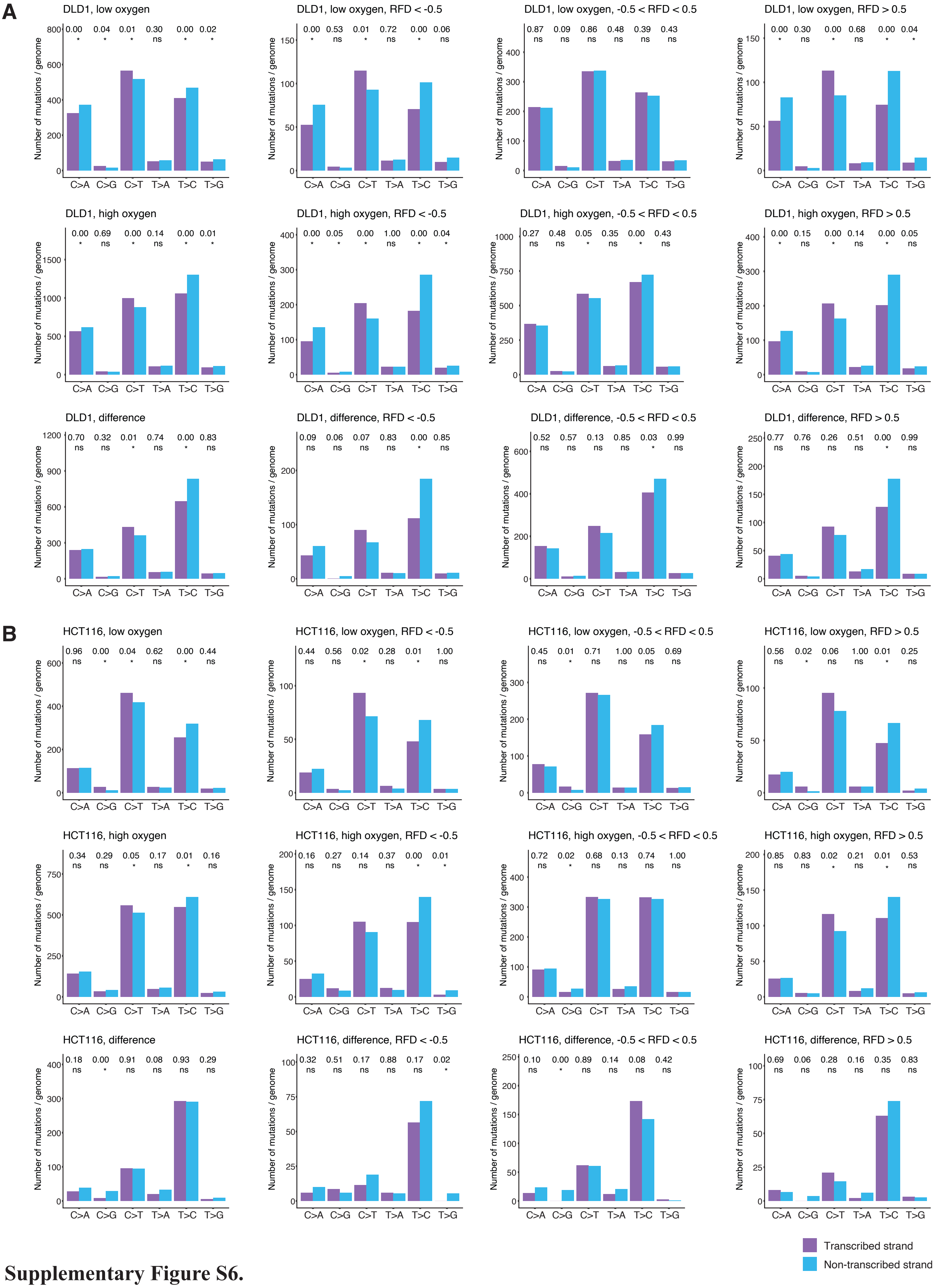

Supplementary Figure S6.

Transcriptional strand bias of SBS mutations observed in genic genomic regions of DLD-1 cells (A) or HCT116 cells (B) kept in low or high oxygen, plotted separately for mutations at the RFD value ranges as indicated on Supplementary Fig. 5B. The statistical significance of the differences is shown above the columns, * $p < 0.05$, ns not significant, χ^2 test. The difference between transcription strand bias in high vs.low oxygen is plotted separately.

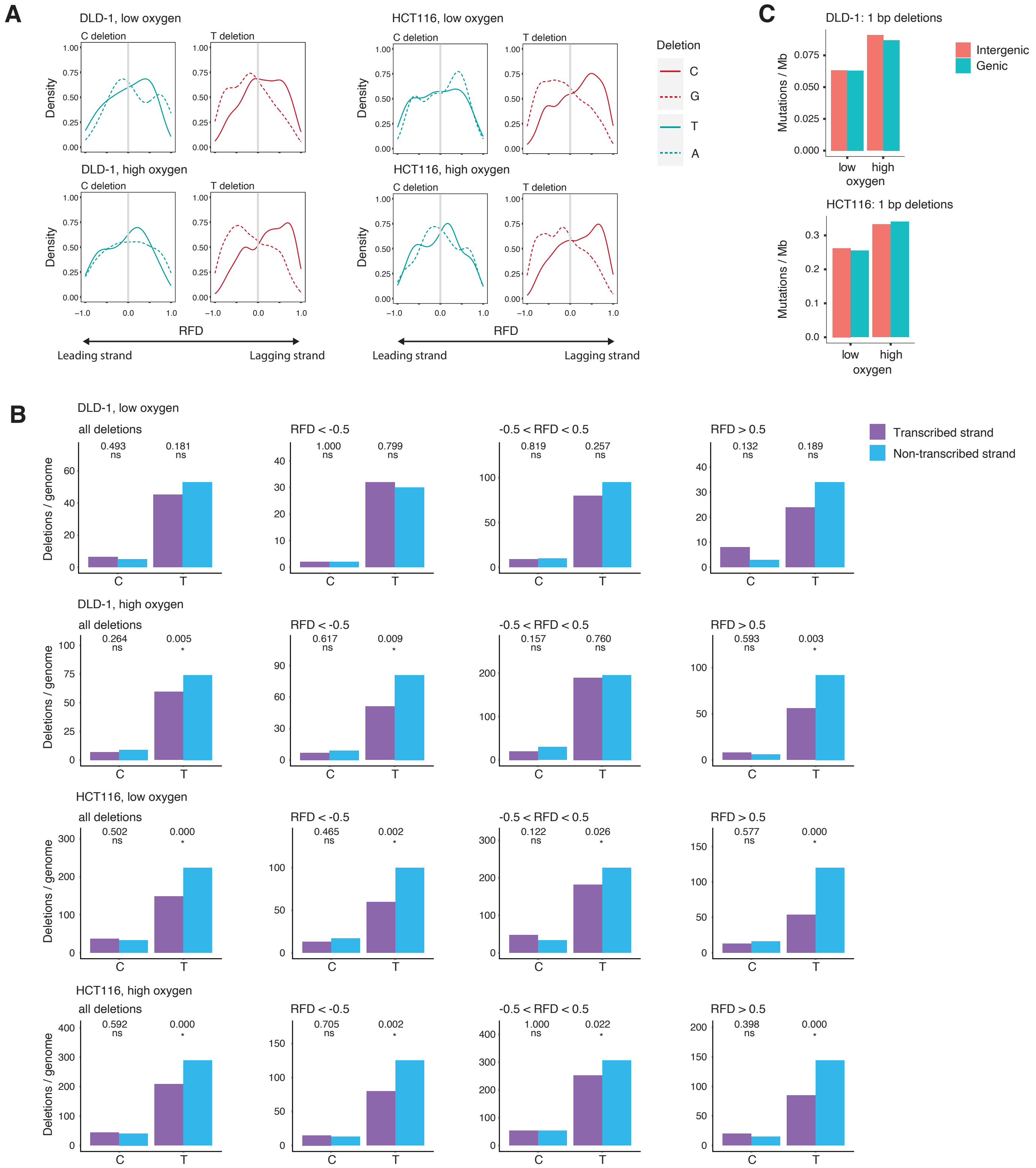

Supplementary Figure S7. Correlation of deletion mutagenesis with genomic features in MMRd cell lines. (A) Distribution of 1 bp deletion classes relative to replication fork directionality (RFD) in DLD-1 or HCT116 cells kept in low or high oxygen. Positive RFD indicates a rightward-oriented fork, in which case the sense strand is the lagging strand. (B) Transcriptional strand bias of deletion mutations observed in genic genomic regions of DLD-1 or HCT116 cells kept in low or high oxygen, plotted separately for mutations at the RFD value ranges as indicated on Supplementary Fig. 5B. The statistical significance of the differences is shown above the columns, * $p < 0.05$, ns not significant, χ^2 test. (C) Mutation densities in genic versus intergenic regions in DLD-1 or HCT116 cells kept in low or high oxygen, shown separately for 1 bp deletions at T or C.

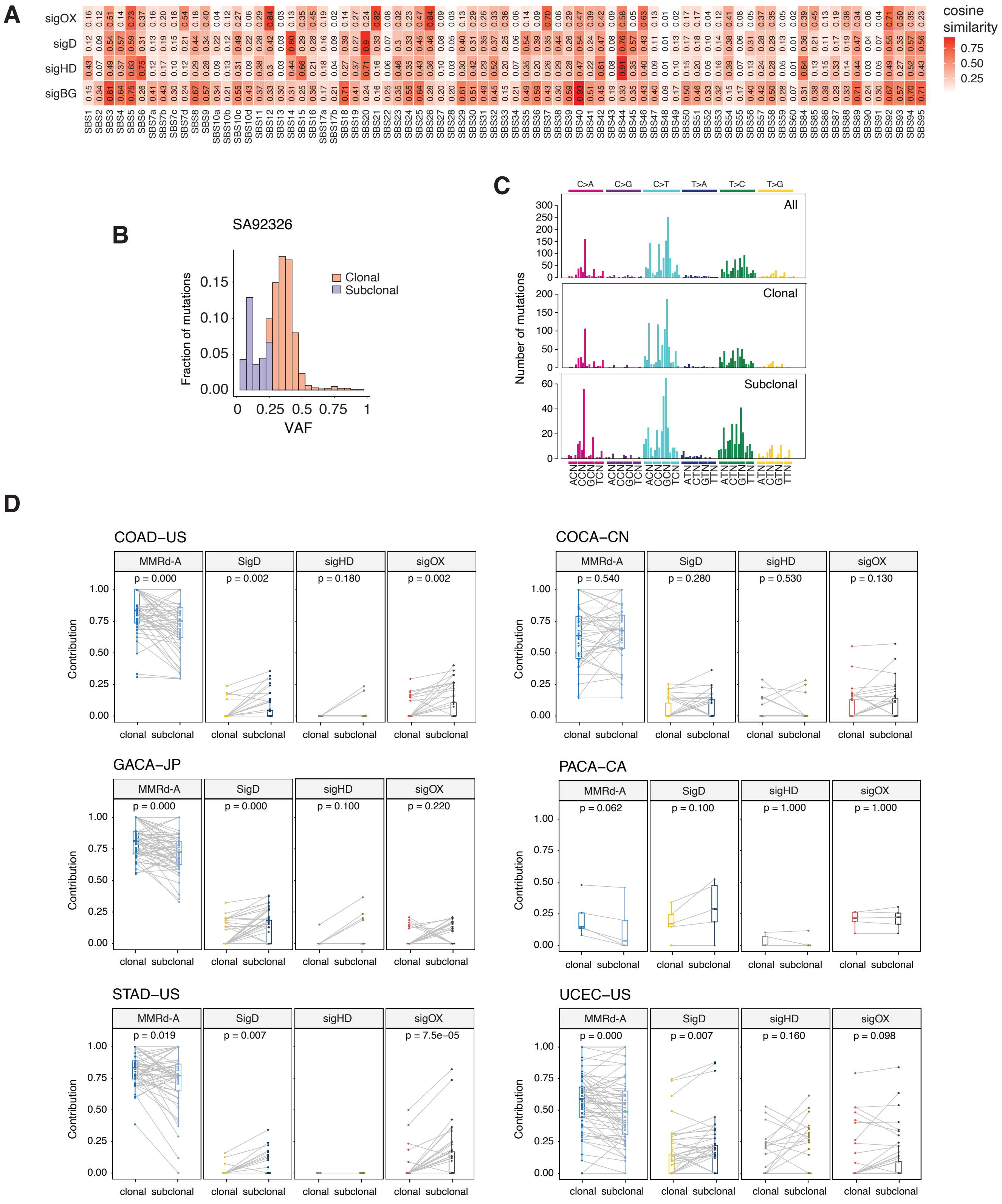

Supplementary Figure S8.

(A) Similarity of the cell line-derived SBS mutation signatures shown in Fig. 6B to COSMIC SBS mutation signatures. (B) An example of the separation of clonal and subclonal mutations based on variant allele frequency (VAF). (C) The triplet SBS mutation spectrum of the total, clonal and subclonal mutation sets of the sample shown in (B). (D) Contribution of the indicated MMRd signatures to the clonal and subclonal mutation sets of MMRd samples of six different PCAWG cohorts deconstructed with the MMRd-substituted COSMIC set. Box plots show the median and the interquartile range; the corresponding clonal and subclonal data points are connected. The significance of the differences is indicated (paired two-sided *t*-tests).

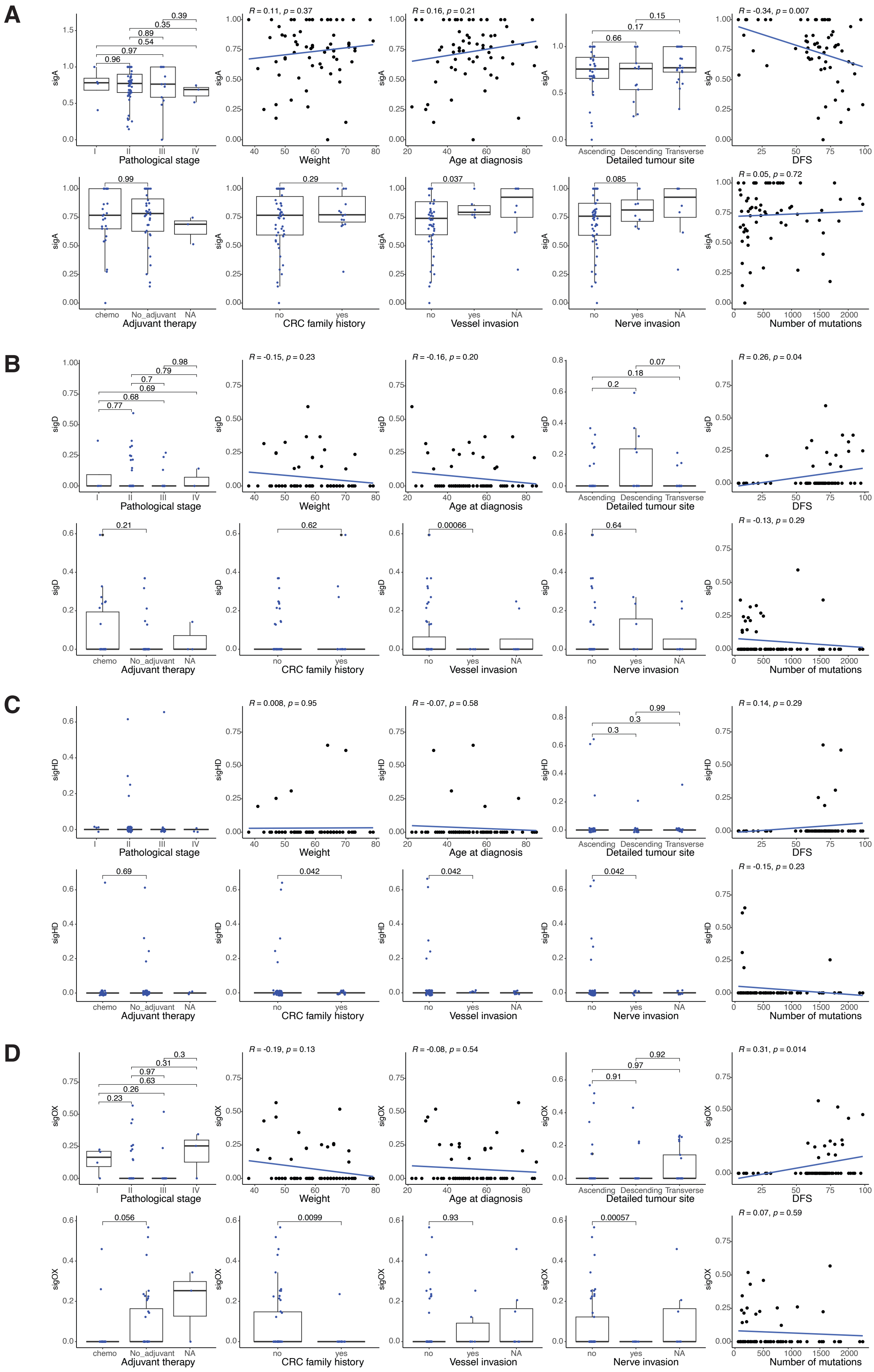

Supplementary Figure S9. Analysis of further colon cancer WES data.
Correlations of selected clinical features with MMRd-A (A), SigD (B), SigHD (C) and SigOX (D) contribution in the deconstruction of subclonal mutational spectra. The significance of the differences is indicated (unpaired two-sided *t*-tests).

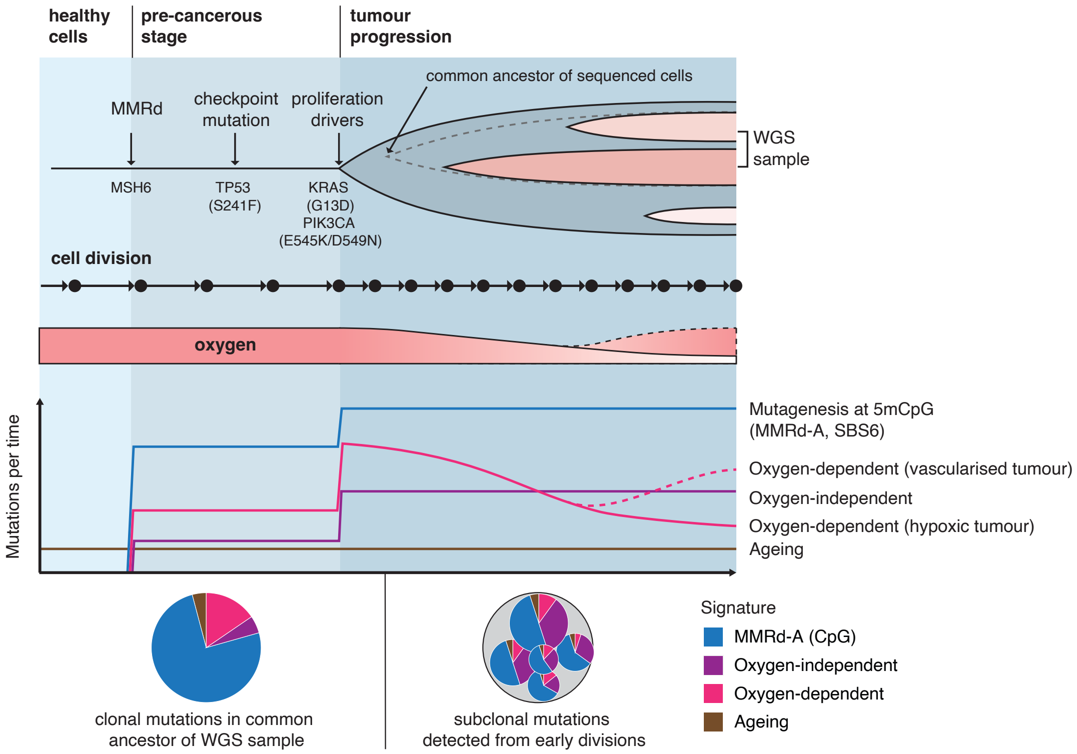

Supplementary Figure S10. A model of mutagenic processes during the evolution of MMRd cancers. The schematic evolution of an MMRd tumour is shown on top, with driver mutations from the colorectal cancer cell line DLD-1 used as an example. Selected subclones are shown. Below, the modelled mutation rates allocated to the indicated mutation signatures are shown in parallel with the inferred cell division rates and oxygen concentration. The loss of MMR function starts a pre-cancerous stage, during which proliferation is not yet increased, thus the mostly replication-independent MMRd-A mutagenesis dominates. Following the acquisition of oncogenic driver mutations such e.g. in KRAS, the proliferation rate increases and mutagenesis attributable to replication-dependent MMRd mutational processes also increases. The balance of oxygen-independent and dependent signatures, better detectable in subclonal mutations, can vary. Two alternative outcomes are shown depending on the oxygen availability of a tumour: hypoxic (solid line) and re-vascularised high oxygen supply (dashed line). Below the main panel, pie charts show the mutation signature contributions to the clonal and subclonal mutation sets of a single theoretical representative MMRd tumour sample.
