# Supplementary material for: DNA mismatch repair protects the genome from oxygen-induced replicative mutagenesis
Source: Nucleic Acids Res. 2023 Oct 4;51(20):11040–55. doi: 10.1093/nar/gkad775 (PMC10639081; doi:10.1093/nar/gkad775)
Supplement: gkad775_Supplemental_Files [file gkad775_supplemental_files.zip › Supplementary_table_S1.pdf]

Supplementary Table 1.

List of WGS samples used for input in IsoMut.

Cell line	Oxygen level	Treatment	File name	ENA ID
DLD-1	low	ancestral clone	DLD1_low_ancestral.bam	ERS12751074
DLD-1	low	mock	DLD1_low_clone1.bam	ERS12751075
DLD-1	low	mock	DLD1_low_clone2.bam	ERS12751076
DLD-1	low	mock	DLD1_low_clone3.bam	ERS12751077
DLD-1	high	ancestral clone	DLD1_high_ancestral.bam	ERS12751068
DLD-1	high	mock	DLD1_high_clone1.bam	ERS12751069
DLD-1	high	mock	DLD1_high_clone2.bam	ERS12751070
DLD-1	high	mock	DLD1_high_clone3.bam	ERS12751071
DLD-1	high	mock	DLD1_high_clone4.bam	ERS12751072
DLD-1	high	mock	DLD1_high_clone5.bam	ERS12751073
TK6	low	ancestral clone	TK6_low_ancestral.bam	ERS12751083
TK6	low	mock	TK6_low_clone1.bam	ERS12751084
TK6	low	mock	TK6_low_clone2.bam	ERS12751085
TK6	low	mock	TK6_low_clone3.bam	ERS12751086
TK6	high	ancestral clone	TK6_high_ancestral.bam	ERS12751078
TK6	high	mock	TK6_high_clone1.bam	ERS12751079
TK6	high	mock	TK6_high_clone2.bam	ERS12751080
TK6	high	mock	TK6_high_clone3.bam	ERS12751081
TK6	high	mock	TK6_high_clone4.bam	ERS12751082
HCT116	low	ancestral clone	HCT116_low_ancestral.bam	ERS15422151
HCT116	low	mock	HCT116_low_clone1.bam	ERS15422152
HCT116	low	mock	HCT116_low_clone2.bam	ERS15422153
HCT116	high	ancestral clone	HCT116_high_ancestral.bam	ERS15422148
HCT116	high	mock	HCT116_high_clone1.bam	ERS15422149
HCT116	high	mock	HCT116_high_clone2.bam	ERS15422150
HCT116+chr3	low	ancestral clone	HCT116_chr3_low_ancestral.bam	ERS15422157
HCT116+chr3	low	mock	HCT116_chr3_low_clone1.bam	ERS15422158
HCT116+chr3	low	mock	HCT116_chr3_low_clone2.bam	ERS15422159
HCT116+chr3	high	ancestral clone	HCT116_chr3_high_ancestral.bam	ERS15422154
HCT116+chr3	high	mock	HCT116_chr3_high_clone1.bam	ERS15422155
HCT116+chr3	high	mock	HCT116_chr3_high_clone2.bam	ERS15422156
DLD-1 *REV1*$^{-/-}$	high	ancestral clone	DLD1_REV1_high_ancestral.bam	ERS16172644
DLD-1 *REV1*$^{-/-}$	high	mock	DLD1_REV1_high_clone1.bam	ERS16172645
DLD-1 *REV1*$^{-/-}$	high	mock	DLD1_REV1_high_clone2.bam	ERS16172646
