# Supplementary material for: DNA mismatch repair protects the genome from oxygen-induced replicative mutagenesis
Source: Nucleic Acids Res. 2023 Oct 4;51(20):11040–55. doi: 10.1093/nar/gkad775 (PMC10639081; doi:10.1093/nar/gkad775)
Supplement: gkad775_Supplemental_Files [file gkad775_supplemental_files.zip › Table S2 File S1 legends.docx]

**Supplementary Table S2.**

Complete list of base substitution (SBS), deletion (DEL) and insertion (INS) mutations found in the whole genome sequenced samples listed in Supplementary Table S1.

**Supplementary File S1.**

Numerical source data, including uncropped gel images, for Figures 1-6.
